# Estimation of left ventricular functions in patients with subclinical hypothyroidism: a meta-analysis

**DOI:** 10.3389/fendo.2023.1279570

**Published:** 2023-12-19

**Authors:** Binyi Li, Yong Huang, Zheng Li

**Affiliations:** ^1^ Department of Ultrasound, The People’s Hospital of Danyang, Danyang Hospital of Nantong University, Danyang, China; ^2^ Department of Endocrinology, The People’s Hospital of Danyang, Danyang Hospital of Nantong University, Danyang, China

**Keywords:** left ventricular function, subclinical hypothyroidism, healthy individuals, practitioners, a meta-analysis

## Abstract

**Objective:**

To evaluate left ventricular (LV) function in patients with subclinical hypothyroidism (ScH) compared to healthy individuals and to provide clinical hints for practitioners.

**Methods:**

PubMed, Embase, Cochrane Library, and Web of Science were systematically searched in this meta-analysis. Studies evaluating LV function in ScH patients were included. Standardized mean difference (SMD) and the 95% confidence intervals (CIs) were calculated as effect size. Heterogeneity and risks of bias of included studies were assessed.

**Results:**

A total of 9 studies were identified as eligible. The SMD for fractional shortening (FS, %) was -0.21 (95% CI: -0.60, 0.17; z = -1.08, p = 0.2788). The pooled SMD for systemic vascular resistance (SVR, dynes/sec·cm-5) was -0.41 (95% CI: -1.31, 0.49; z = -0.89, p = 0.3744). The pooled SMD for early diastolic mitral flow velocity/late diastolic mitral flow velocity (E/A) ratio was -0.74 (95% CI: -1.09, -0.39; z = -4.13, p < 0.001). The pooled SMD for ejection fraction (EF, %) was -0.35 (95% CI: –0.59, -0.12; z = -2.95, p = 0.0032).

**Conclusion:**

ScH patients had significantly worse LV function parameters than healthy controls. These changes in LV function may be involved in the management of ScH.

## 1 Introduction

Subclinical hypothyroidism (ScH) is a condition in which the concentration of serum thyroid-stimulating hormone (TSH) slightly increases and the levels of circulating thyroid hormones, free thyroxine (FT4), and triiodothyronine (FT3) remain normal (1). ScH is a relatively common disease, and most population-based studies have shown a prevalence rate of approximately 5-10% of the population (1–4). Unlike overt hypothyroidism, patients with ScH do not necessarily demonstrate clinical features of hypothyroidism and their condition is usually detected through routine health examinations (5). The normal range of serum TSH may vary by country or ethnicity (6). Moreover, age is another important factor affecting TSH levels, which tend to increase with age, meaning that a mild increase may be normal for older people (7). Therefore, most guidelines recommend developing a reference range for serum TSH based on population, age, and trimester (1, 8).

The impact of ScH on the cardiovascular system has been extensively studied for the past three decades (9). Due to the relative deficiency of thyroid hormones, ScH may include some symptoms of hypothyroidism and different cardiovascular events (10). In their study, Biondi et al. have shown that even subclinical forms of autoimmune thyroid disease can affect cardiac function (11). A meta-analysis of nearly 60000 participants reported an increased risk of fatal and non-fatal events such as coronary heart disease, congestive heart failure, and fatal stroke in individuals with elevated TSH levels (12–14). Recently, it has been proposed that ScH is related to atrial dysfunction and atrial fibrillation in experimental animal models (15, 16). Although thyroid hormonal therapy is effective, there is still no consensus on the timely identification of myocardial involvement in asymptomatic ScH patients and the response to clinical treatment, which may be due to the lack of reliable indicators of cardiac involvement and the functional heterogeneity of these patients (17). Of note, most existing studies focused on the structure and function of the LV, using pulse and tissue Doppler imaging to estimate LV function (18–22). Mechanical abnormalities of the LV may lead to increased morbidity, exercise intolerance, and heart failure (23). In ScH patients, cardiac anomalies are more common at higher TSH levels and among older individuals (18). Previous studies have shown that patients with ScH have prolonged Isovolumic Relaxation Time (IRT), increased A wave, and decreased early diastolic mitral flow velocity/late diastolic mitral flow velocity (E/A) ratio (18). In addition, the Cardiovascular Health Study revealed that patients >_65 years with TSH≥10 mIU/L had higher baseline peak E velocity (0.80 m/s) than those with normal TSH (0.72 m/s, P = 0.002) (24).

There is still no meta-analysis based on published studies that investigated the relationship between ScH and LV function among untreated patients with ScH. Accordingly, this meta-analysis evaluated LV function in patients with ScH, providing possible clinical indications for practitioners.

## 2 Methods

The meta-analysis was done referring to the Preferred Reporting Items for Systematic Reviews and Meta-analysis (PRISMA) (25). All the original articles received Ethical approvals, so no ethical approval was required for this meta-analysis.

### 2.1 Search strategy and selection criteria

Two independent authors comprehensively searched the electronic databases, including PubMed, Embase, Cochrane Library, and Web of Science, from their inception to June 8, 2023. Only articles in the English language were considered. The search strategies for online databases are presented in **Table S1**. Inclusion criteria were as follows: 1) study population included patients with ScH; 2) controlled studies; 3) studies that included outcomes concerning LV function reported in both ScH group and control group, or the possibility to calculate indicators mentioned above. Exclusion criteria were: 1) data could not be extracted or calculated; 2) case reports or series, reviews, conference abstracts, non-English articles, and animal studies; 3) duplicates or overlap in the research participants. Two researchers independently carried out the database search and study selection. Disagreements were resolved through discussion.

### 2.2 Data extraction and quality assessments

Two independent authors screened the title and abstract of citations in the first round of study selection, after which a full-text assessment of the studies was conducted for the final inclusion. Besides the relevant outcomes mentioned above, we extracted the following parameters: the name of the first author, year of publication, age (year), percentage of females, FT4 levels, FT3 levels, TSH levels in CsH patients and control, outcomes on LV. The specific LV parameters to be pooled depended on the number of included studies in each parameter. Changes in LV parameters of ScH patients after and before treatment were also assessed. The Cochrane Collaboration’s tool for assessing the risk of bias was used to evaluate the risk of bias for randomized controlled trials (RCTs), and the Newcastle-Ottawa scale (NOS) criteria was used for non-randomized studies (26, 27).

### 2.3 Statistical analysis

R language and environment were used for statistical computing (R Foundation for Statistical Computing, Vienna, Austria; version 4.1.2) data analyses. Standardized mean difference (SMD) and the 95% confidence intervals (CIs) were calculated as endpoints. An SMD value > 0 represented the superior effect in ScH patients over control; otherwise, an SMD value < 0 indicated the inferior effect of ScH over control, and an SMD value = 0 suggested an equivalent effect in both ScH and control groups. The I^2^ statistics were used to assess the heterogeneity between the included studies. Insignificant, low, moderate, and high heterogeneity were graded as I^2^ values of 0 -, 25% -, 50% -, and 75 - 100%, respectively (28). Funnel plots and Egger’s test for asymmetry of the funnel plots were performed to assess the publication bias of included studies. In addition, sensitivity analysis was performed to evaluate the impact of a single study on the overall outcomes. A p-value < 0.05 was considered to be statistically significant.

## 3 Results

### 3.1 Study selection and characteristics

**Figure 1:**
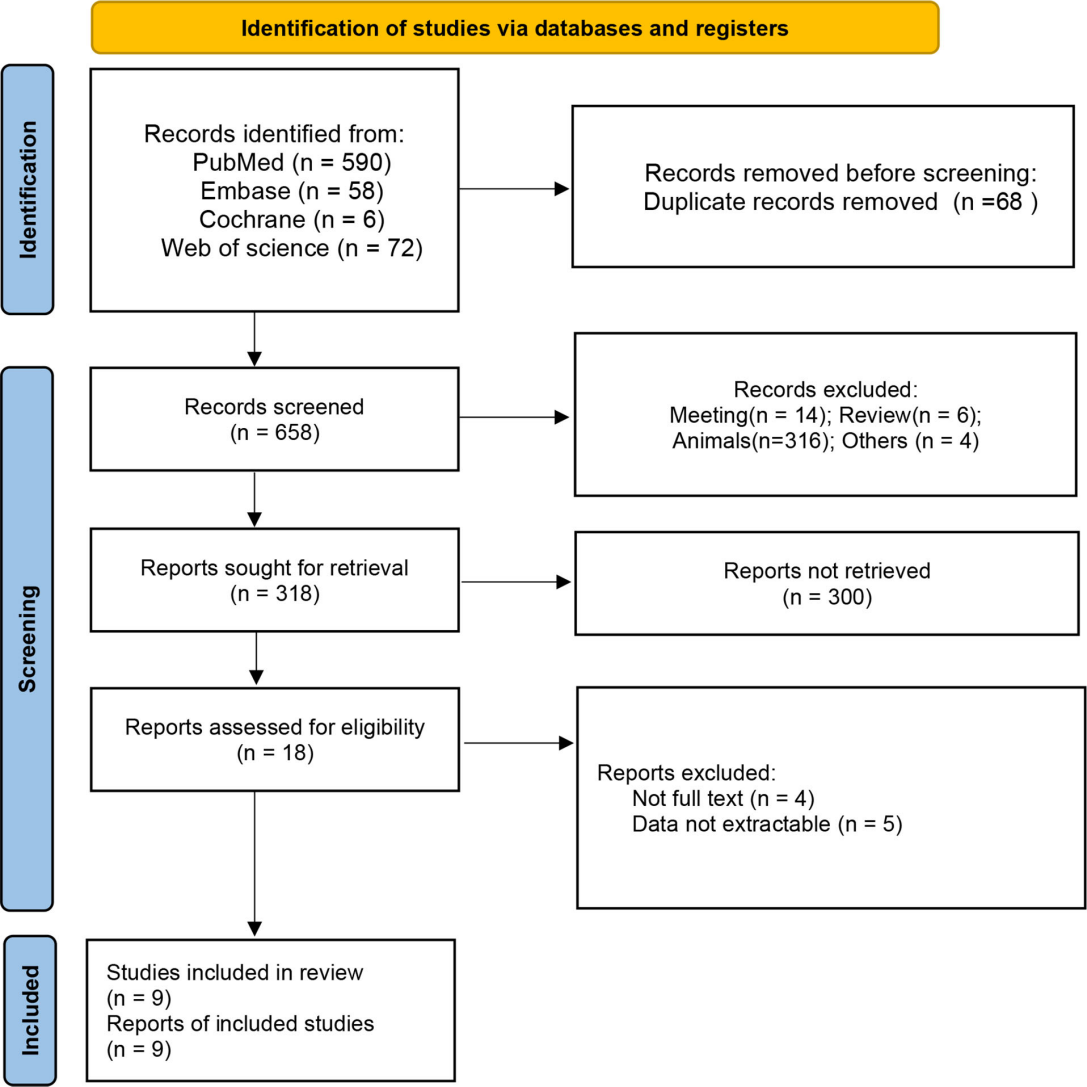
Flowchart of the literature search.

A total of 726 articles were identified from the databases searched. Following an initial screening of these citations, 68 duplicates and 640 ineligible studies were eliminated. After a full-text assessment for eligibility of the remaining 18 citations, 9 studies were identified for inclusion in this meta-analysis (10, 18–22, 29–31). No additional studies were identified through screening of references of related reviews (**Figure 1**). All included studies were non-randomized controlled studies, and they included a total of 661 patients with Sch and 4026 controls, where the majority of participants were female. Six studies reported that the etiology of ScH was autoimmune thyroiditis, while the remaining 3 studies did not specify the cause of ScH (**Table 1**). Other accompanying diseases were not mentioned. The overall quality of the included studies was rated as high based on the NOS criteria (**Table 2**).

**Table 1:** Study characteristics.

Author	Year	Diagnosis of ScH	Treatment	Etiology of ScH	Equipment used for LV function assessment	ScH patients						Control					
						n	Age (yr)	Female (%)	FT4 (pmol/L)	FT3 (pmol/L)	TSH (mU/L)	n	Age (yr)	Female (%)	FT4 (pmol/L)	FT3 (pmol/L)	TSH (mU/L)
Biondi	1999	TSH values above normal, associated with a supranormal response to TRH (ΔTSH above 30 mU/L), and FT3 and FT4 in the lower limit of the normal range	L-T_4_ treatment	NS	Complete mono- and two-dimensional, and Doppler-echocardiography	26	36 ± 12	92	9.4 ± 3.0	5.1 ± 1.1	8.6 ± 4.8	30	36 ± 11	80	15.3 ± 2.6	6.0 ± 1.2	1.6 ± 0.9
Ripoli	2005	4.00<TSH<15 μIU/ml, in the presence of both FT_3_ and FT_4_ in the normal range	substitutive treatment with synthetic thyroid hormone	autoimmune thyroiditis	whole-body magnetic resonance imaging	30	41.2 ± 6.9	NS	14.8 ± 4	4.9 ± 0.8	8.7 ± 3.7	20	38.4 ± 9.4	NS	15.3 ± 2	5.2 ± 0.6	2.3 ± 0.6
Zoncu	2005	serum TSH >3mU/ml, FT_3_ concentration was within the normal range	NA	Hashimoto’s thyroiditis	pulsed wave tissue Doppler imaging	32	42 ± 10.4	97	11.2 ± 2.6	4.9 ± 0.8	4.4 ± 2.3	13	39.0 ± 8.2	92	13.3 ± 2.2	5.4 ± 0.3	1.2 ± 0.5
Aghini-Lombardi	2006	serum TSH level ranging between 3.5 and 7.5 mU/L	NA	autoimmune thyroiditis	two-dimensional (2D) color Doppler echocardiography, pulsed wave tissue Doppler imaging (PWTDI)	24	34.8 ± 6.2	NS	11.6 ± 2.3	5.4 ± 1.1	5.3 ± 1.1	24	33.9 ± 6.4	NS	15.9 ± 3.5	6 ± 5.2	1.4 ± 0.8
Tadic	2014	serum TSH level (>5 mIU/mL) with normal levels of FT_3_ and FT_4_	levothyroxine therapy	autoimmune thyroiditis	two-dimensional (2D) and 3D color Doppler echocardiography	54	41 ± 6	NS	14 ± 2.1	2.8 ± 0.6	8.8 ± 2.7	40	40 ± 7	NS	14.8 ± 2.9	3 ± 0.5	2.3 ± 0.9
Chen	2016	increased serum TSH level with normal levels of serum FT_3_ and FT_4_	levothyroxine therapy	autoimmune thyroiditis	Three-Dimensional Speckle Tracking Echocardiography	32	40.7 ± 9.7	63	17.7 ± 6.2	5 ± 1.8	5.6 ± 1.3	30	38.7 ± 8.2	67	19.4 ± 4.4	5.1 ± 1.9	3.5 ± 1.2
Nakova	2018	4.2 mU/L < TSH < 10.0 mU/L with normal serum FT_4_ 10.3–24.45 pmol/L and FT_3_ 4.2–8.1 pmol/L.	levothyroxine therapy	NS	Twodimensional (2D) echocardiography, pulsed wave (PW) Doppler, tissue Doppler imaging (TDI), and 2D speckle tracking echocardiography	54	43.1 ± 12.4	96	12.3 ± 2.0	4.5 ± 1.1	8.1 ± 1.3	30	39.3 ± 11.7	90	12.3 ± 2.0	4.5 ± 1.1	8.1 ± 1.3
Huang	2021	NS	NA	NS	tissue Doppler imaging (TDI) and speckle-tracking-based strain analysis	374	52.2 ± 12.4	56	NS	NS	6 ± 3.2	3799	48.8 ± 11.2	34	NS	NS	1.9 ± 0.8
Pandrc	2021	TSH between the upper limit of normal (4 mIU/L) and 10 mIU/L, normal FT4, positive thyroid antibodies (TPO Ab) and/or an ultrasound scan consistent with chronic autoimmune thyroiditis	L-T_4_ treatment	autoimmune thyroiditis	3D strain echocardiography	35	51.6 ± 15.4	83	13.4	1.7 ± 0.2	6.9 ± 2.1	40	47.3 ± 13.1	80	NS	NS	NS

NS, not specified. NA, not applicable.

**Table 2:** NOS criteria for quality of non-randomized controlled studies.

Study	Representativeness of the exposed cohort	Selection of the non-exposed cohort	Ascertainment of exposure	Demonstration that the outcome of interest was not present at the start of the study	Comparability of cohorts on the basis of the design or analysis	Assessment of outcome	Was follow-up long enough for outcomes to occur	Adequacy of follow-up of cohorts	Total quality scores
Biondi 1999	*	*		*	**	*	*	NA	7
Ripoli 2005	*	*		*	**	*	*	NA	7
Zoncu 2005	*	*		*	**	*	*	NA	7
Aghini-Lombardi 2006	*	*		*	**	*	*	NA	7
Tadic 2014	*	*		*	**	*	*	NA	7
Chen 2016	*	*		*	**	*	*	NA	7
Nakova 2018	*	*		*	**	*	*	NA	7
Huang 2021	*	*		*	**	*	*	NA	7
Pandrc 2021	*	*		*	**	*	*	NA	7

* stand for the score of NOS, the maximum score on the NOS is 9 (highest quality), and we assigned scores of 0–3, 4–6, and 7–9 for low, moderate, and high quality of studies, respectively. NA, not applicable.

### 3.2 LV function parameters before treatment

**Figure 2:**
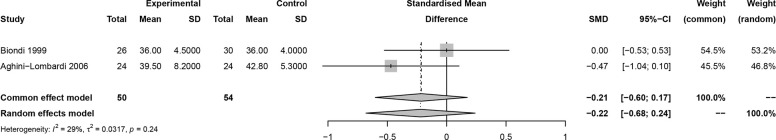
Forest plot of fractional shortening.

**Figure 3:**
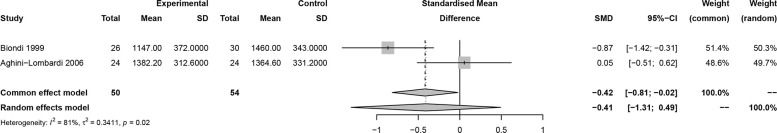
Forest plot of systemic vascular resistance.

**Figure 4:**
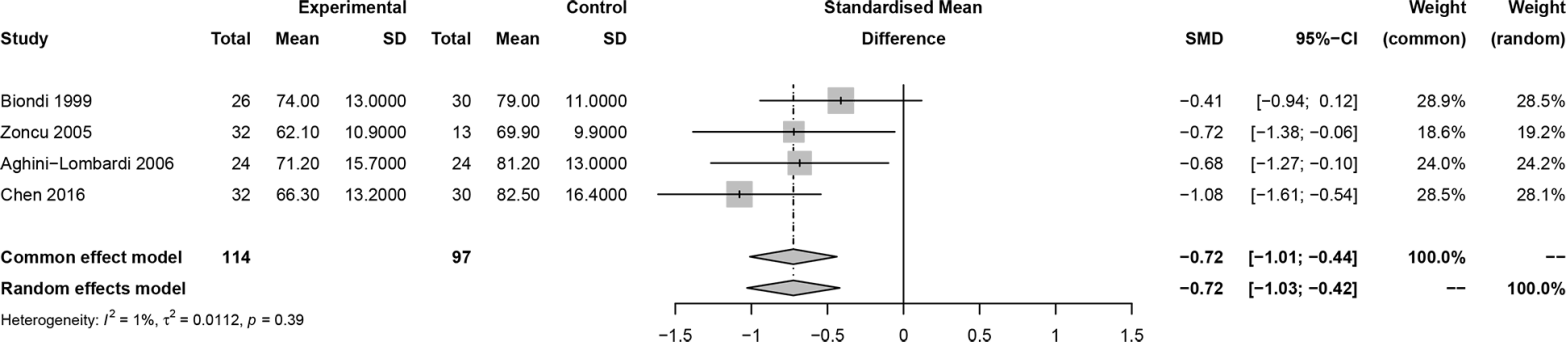
Forest plot of early diastolic mitral flow velocity.

**Figure 5:**
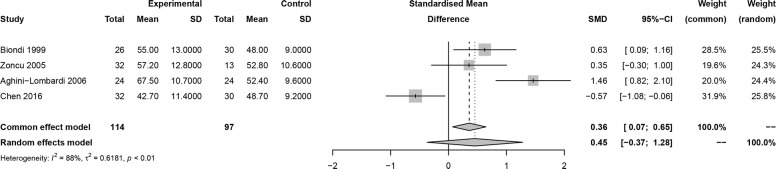
Forest plot of late diastolic mitral flow velocity.

**Figure 6:**
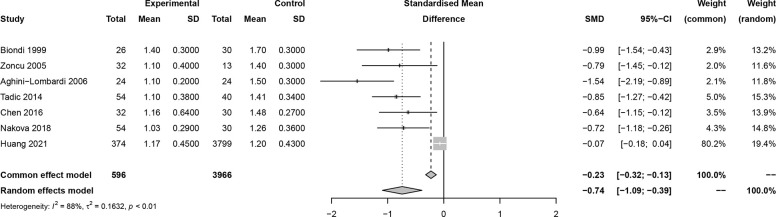
Forest plot of early diastolic mitral flow velocity/late diastolic mitral flow velocity (E/A) ratio.

**Figure 7:**
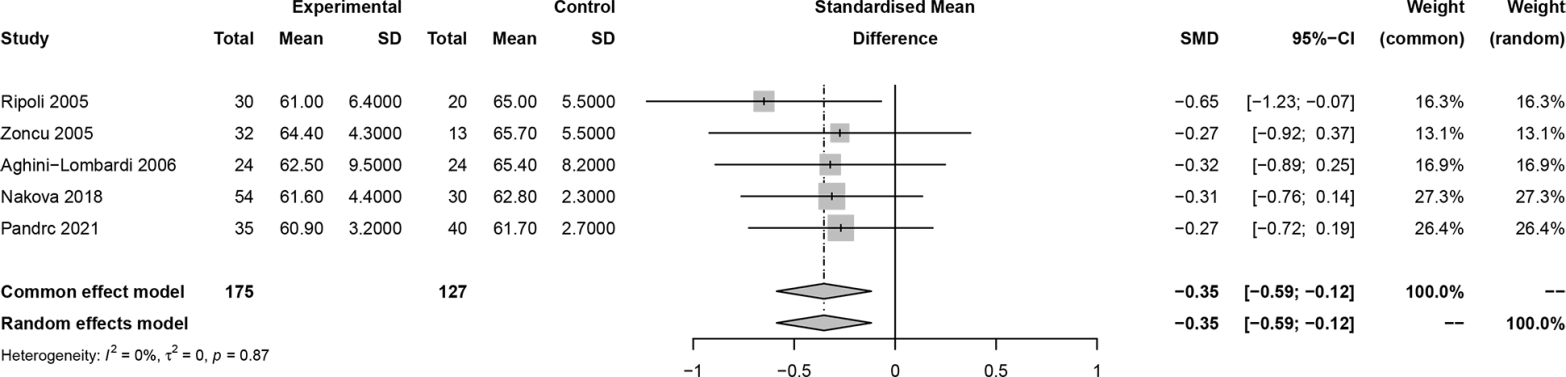
Forest plot of ejection fraction.

In this meta-analysis, 2 studies reported outcomes on fractional shortening (FS, %), and the SMD of −0.21 (95% CI: -0.60, 0.17; z = -1.08, p = 0.2788) (**Figure 2**). Two studies reported results on systemic vascular resistance (SVR, dynes/sec·cm-5), where the pooled SMD was -0.41 (95% CI: -1.31, 0.49; z = -0.89, p = 0.3744) (**Figure 3**). Four studies reported outcomes on early diastolic mitral flow velocity (cm/sec), and the pooled SMD of -0.72 (95% CI: -1.01, -0.44; z = -4.97, p < 0.001) (**Figure 4**). Four included studies reported late diastolic mitral flow velocity (cm/sec), where the pooled SMD was 0.45 (95% CI: -0.37, 1.28; z = 1.08, p = 0.2797) (**Figure 5**). Seven studies reported an E/A ratio, where the pooled SMD was -0.74 (95% CI: -1.09, -0.39; z = -4.13, p < 0.001) (**Figure 6**). Five studies reported results on ejection fraction (EF, %), where the pooled SMD was -0.35 (95% CI: –0.59, -0.12; z = -2.95, p = 0.0032) (**Figure 7**). The SMD for HR, SBP, and DBP were -0.05 (95% CI: -0.15, 0.04; z = -1.14, p = 0.2537), 0.19 (95% CI: -0.07, 0.45; z = 1.43, p = 0.1535), and -0.03 (95% CI: -0.12, 0.06; z = -0.71, p = 0.4799) (**Table 3**). Four studies reported results on global longitudinal strain (GLS), where the pooled SMD was 0.70 (95% CI: 0.47, 0.93; z = 5.91, p < 0.001) (**Table 3**). Three studies reported results on global circumferential strain (GCS), where the pooled SMD was 0.65 (95% CI: 0.38, 0.92; z = 4.77, p < 0.001) (**Table 3**). End-diastolic volume (EDV) was lower in ScH patients than in controls (64.3 ± 8.7 ml/m^2^ *vs*. 81.4 ± 11.3 ml/m^2^, p < 0.001) in Ripoli’s study (19). Significant prolongation of the isovolumic relaxation time (94 ± 13 *vs*. 84 ± 8 msec; p < 0.001) and increased A wave (55 ± 13 *vs*. 48 ± 9 cm/sec; p < 0.05) were observed in the ScH patients as compared to the controls in the study reported by Biondi et al. (18). In Chen’s study, ScH patients had significantly lower global mechanical index (GMI) compared with the controls (-386.3 ± 48.6 *vs*. - 630.8 ± 64.8, p < 0.0001) (22).

**Table 3:** Results of pooled analyses on LV parameters.

LV function parameters	No. studies	SMD	95% CI	I^2^ (%)	z	p
HR	8	-0.05	-0.15, 0.04	8	-1.14	0.2537
SBP	9	0.19	-0.07, 0.45	67	1.43	0.1535
DBP	9	-0.03	-0.12, 0.06	2	-0.71	0.4799
EDD	5	0.03	-0.19, 0.25	32	0.28	0.7830
ESD	3	0.17	-0.11, 0.44	0	1.19	0.2336
IVST	5	0.22	-0.04, 0.47	61	1.67	0.0951
LVPWTD	6	0.26	-0.01, 0.52	66	1.92	0.0548
GLS	4	0.70	0.47, 0.93	36	5.91	< 0.0001
GCS	3	0.65	0.38, 0.92	0	4.77	< 0.0001

HR, heart rate; SBP, systolic blood pressure; DBP, diastolic blood pressure; EDD, end-diastolic diameter; ESD, end-systolic diameter; IVST, interventricular septum thickness; LVPWTD, left ventricle posterior wall thickness; GLS, global longitudinal strain; GCS, global circumferential strain.

### 3.3 LV function parameters after *vs*. before treatment

Six studies reported changes in LV functions after and before treatment in ScH patients (10, 18, 19, 22, 29, 30). EF was significantly increased after treatment in ScH patients [SMD: 0.55 (95% CI: 0.18, 0.91; z = 2.92, p = 0.0035)] (**Table 4**). GLS and GCS were significantly reduced after treatment [GLS: SMD: -0.47 (95% CI: -0.77, -0.18; z = -3.12, p = 0.0018); GLS: SMD: -0.37 (95% CI: -0.66, -0.07; z = -2.43, p = 0.0150)] (**Table 4**). Other changes in LV parameters are listed in **Table 4**.

**Table 4:** Results of pooled analyses on LV parameters before and after treatment.

LV function parameters	No. studies	SMD	95% CI	I^2^ (%)	z	p
E/A ratio	4	0.52	0.11, 0.92	64	2.49	0.0128
EF	4	0.55	0.18, 0.91	58	2.92	0.0035
EDD	3	-0.09	-0.33, 0.14	0	-0.78	0.4345
ESD	2	-0.21	-0.51, 0.09	0	-1.38	0.1673
IVST	3	-0.12	-0.36, 0.11	0	-1.04	0.3003
LVPWTD	4	-0.06	-0.28, 0.15	0	-0.58	0.5628
GLS	2	-0.47	-0.77, -0.18	0	-3.12	0.0018
GCS	2	-0.37	-0.66, -0.07	0	-2.43	0.0150

E/A ratio, early diastolic mitral flow velocity/late diastolic mitral flow velocity; EF, ejection fraction (%); EDD, end-diastolic diameter; ESD, end-systolic diameter; IVST, interventricular septum thickness; LVPWTD, left ventricle posterior wall thickness; GLS, global longitudinal strain; GCS, global circumferential strain.

### 3.4 Publication bias

Insignificant publication bias was detected based on linear regression tests of funnel plot asymmetry on early diastolic mitral flow velocity (p = 0.9851), late diastolic mitral flow velocity (p = 0.2952), and EF (p = 0.5553), EDD (p = 0.8529), IVST (p = 0.1913), LVPWT (p = 0.0780), GLS (p = 0.4056), GCS (p = 0.9663), HR (p = 0.4511), SBP (p = 0.2606), DBP (p = 0.4127) except for E/A ratio (p = 0.0007), and ESD (p = 0.0321) (**Figures S1**-**13**).

### 3.5 Sensitivity analysis

Results on sensitivity analyses revealed that most pooled results are relatively stable after removing one study after another (**Figures S14**-**36**).

## 4 Discussion

This meta-analysis focused on LV functions of untreated patients with ScH. Outcomes on FS, SVR, early diastolic mitral flow velocity, late diastolic mitral flow velocity, E/A ratio, EF, EDD, ESD, IVST, LVPWT, GLS, and GCS were analyzed according to the actual number of included studies.

Results demonstrated that early diastolic mitral flow velocity was significantly lower in ScH patients than in the control group; the E/A ratio was also significantly decreased in the ScH group compared to the control group. GLS and GCS were significantly increased in ScH patients compared to the controls. Our findings are consistent with some previous studies (10, 29). A decrease in peak E mitral flow velocity, an increase in peak A mitral flow velocity, and a decrease in E/A ratio were detected in the ScH group compared to the control in the study by Aghini-Lombardi et al. (21). In the study of Biondi et al., a reduced E/A ratio was detected in the ScH group compared to the control (1.4 ± 0.3 *vs*. 1.7 ± 0.3; P < 0.001) (18), which suggested initial impairment of left ventricular diastolic function among ScH patients. In their study, Di Bello et al. have shown that ScH is associated with early changes in myocardial function and structure via routine echocardiography and video densitometer analysis (32). The biological changes in the myocardium can cause left ventricular diastolic dysfunction (33). A slight decrease in hormone activity at the myocardial level in ScH patients may lead to biochemical and functional effects, including decreased heart rate and impairment of myocardial contraction and relaxation, similar to overt hypothyroidism, which is the cause of changes in myocardial function (34, 35). Moreover, this study showed that EF was significantly lower in ScH patients than in controls, which was consistent with previous single studies failing to reach statistical significance (10, 19–21, 30). These conflicting results may be explained by different selections of patients (number, age, type, and length of ScH), different diagnostic criteria (range of TSH levels), and different techniques and indexes used to assess LV function. The underlying mechanism for ScH on the systolic LV performance measured by EF needs to be investigated further. Moreover, GLS and GCS were significantly reduced after treatment in this meta-analysis. Of note, only 2 studies reported outcomes on GLS and GCS before and after treatment in ScH patients, so these findings should be interpreted with caution. However, it is important to note that the reduction in left ventricular systolic and diastolic function in patients with ScH is still within the normal range of the left ventricle itself, which can lead clinicians to overlook the impact of ScH on left ventricular function in their clinical care and in some studies. Many patients with ScH can benefit from treatment, and therefore guidelines commissioned by the American Association of Clinical Endocrinologists (AACE) in conjunction with the American Thyroid Association (ATA) recommend that patients with ScH with a TSH greater than 10 mIUL should be treated (36).

The quality of the included studies was rated as high according to the NOS criteria. Insignificant publication bias was detected except for the analysis of the E/A ratio and ESD. Results of this study provide high-grade evidence on left ventricular function in patients with ScH and hints for the early detection of Sch through LV function tests.

There are some limitations to this meta-analysis. Firstly, non-randomized controlled studies were included in this study; therefore, the causal relationship could not be proven. Accordingly, more well-designed studies, primarily randomized controlled trials (RCTs), are warranted in the future to address this issue. Secondly, different techniques were utilized to assess LV function, which may be another source of heterogeneity between enrolled studies. Thirdly, individual patient data cannot be obtained, and the impact of other covariates on the overall outcomes could not be assessed. Fourthly, although we followed the parameters of LV systolic and diastolic function, only 6 parameters were analyzed in the present study, while other related parameters were not pooled due to the limited number of studies reporting on those indicators; in addition, the severity of ScH was not assessed in this meta-analysis because no related data could be retrieved from the included studies, more relevant studies are needed for a more comprehensive analysis of the LV function.

Based on the findings of this study, we may conclude that patients with ScH have worse changes in certain parameters indicative of the involvement of systolic and diastolic function of the LV in ScH compared to healthy individuals. Results on LV function may assist practitioners in the management of ScH.

## Author contributions

BL: Data curation, Formal Analysis, Writing – original draft, Writing – review & editing. YH: Data curation, Formal Analysis, Writing – original draft. ZL: Data curation, Formal Analysis, Writing – review & editing.
