# Supplementary material for: Estimation of left ventricular functions in patients with subclinical hypothyroidism: a meta-analysis
Source: Front Endocrinol (Lausanne). 2023 Dec 19;14:1279570. doi: 10.3389/fendo.2023.1279570 (PMC10763245; doi:10.3389/fendo.2023.1279570)
Supplement: Supplementary file 1 [file DataSheet_1.docx]

**Supplementary Table 1 Search strategy for online databases.**

**Search strategy of PubMed**

| NO. | Search Details | Results |
| --- | --- | --- |
| #6 | (#1 OR #3) AND (#2 OR #4 OR #5) | 590 |
| #5 | (((((((((((Left ventricular strain) OR (Left ventricular deformation)) OR (Left ventricular myocardial strain)) OR (Myocardial deformation imaging)) OR (Speckle tracking echocardiography)) OR (Longitudinal strain)) OR (Circumferential strain)) OR (Radial strain)) OR (3D strain)) OR (Regional strain)) OR (Strain rate)) OR (Strain imaging) | 385,323 |
| #4 | (((((((((((((((Global Longitudinal Strains) OR (Global Longitudinal Strain)) OR (Left Ventricular Global Longitudinal Strain)) OR (LV-GLS)) OR (LV GLS)) OR (global left ventricle longitudinal strain (GLS))) OR (left ventricle longitudinal strain)) OR (left ventricle longitudinal strain (LV LS))) OR (left ventricular (LV) global longitudinal strain (GLS))) OR (left ventricular global longitudinal strain (LV-GLS))) OR (left ventricular global longitudinal strain (LVGLS))) OR (left ventricular GLS)) OR (LV global longitudinal strain)) OR (LV global longitudinal strain (GLS))) OR (LV longitudinal global strain (GLS))) OR (LV longitudinal global strain (LV-GLS)) | 7,227 |
| #3 | (((((((((((((((((((((acute hypothyroidism) OR (Central Hypothyroidism)) OR (Central Hypothyroidisms)) OR (hypothyreoidism)) OR (hypothyreosis)) OR (hypothyroidea)) OR (hypothyroidism)) OR (Hypothyroidisms)) OR (hypothyroidosis)) OR (hypothyrosis)) OR (primary hypothyroidism)) OR (Primary Hypothyroidisms)) OR (Secondary Hypothyroidism)) OR (Secondary Hypothyroidisms)) OR (thyroid deficiency)) OR (thyroid gland failure)) OR (thyroid insufficiency)) OR (Thyroid Stimulating Hormone Deficiency)) OR (Thyroid-Stimulating Hormone Deficiencies)) OR (Thyroid-Stimulating Hormone Deficiency)) OR (TSH Deficiencies)) OR (TSH Deficiency) | 64,935 |
| #2 | "Global Longitudinal Strain"[Mesh] | 37 |
| #1 | "Hypothyroidism"[Mesh] | 35,066 |

**Search strategy of EMBASE**

| No. | Query | Results |
| --- | --- | --- |
| #8 | #6 AND #7 | 58 |
| #7 | #3 OR #4 OR #5 | 31803 |
| #6 | #1 OR #2 | 99926 |
| #5 | 'left ventricular strain':ti,ab,kw OR 'left ventricular deformation':ti,ab,kw OR 'left ventricular myocardial strain':ti,ab,kw OR 'myocardial deformation imaging':ti,ab,kw OR 'speckle tracking echocardiography':ti,ab,kw OR 'longitudinal strain':ti,ab,kw OR 'circumferential strain':ti,ab,kw OR 'radial strain':ti,ab,kw OR '3d strain':ti,ab,kw OR 'regional strain':ti,ab,kw OR 'strain rate':ti,ab,kw OR 'strain imaging':ti,ab,kw | 31522 |
| #4 | 'global longitudinal strains':ti,ab,kw OR 'global longitudinal strain':ti,ab,kw OR 'left ventricular global longitudinal strain':ti,ab,kw OR 'lv-gls':ti,ab,kw OR 'lv gls':ti,ab,kw OR 'global left ventricle longitudinal strain (gls)':ti,ab,kw OR 'left ventricle longitudinal strain':ti,ab,kw OR 'left ventricle longitudinal strain (lv ls)':ti,ab,kw OR 'left ventricular (lv) global longitudinal strain (gls)':ti,ab,kw OR 'left ventricular global longitudinal strain (lv-gls)':ti,ab,kw OR 'left ventricular global longitudinal strain (lvgls)':ti,ab,kw OR 'left ventricular gls':ti,ab,kw OR 'lv global longitudinal strain':ti,ab,kw OR 'lv global longitudinal strain (gls)':ti,ab,kw OR 'lv longitudinal global strain (gls)':ti,ab,kw OR 'lv longitudinal global strain (lv-gls)':ti,ab,kw | 10602 |
| #3 | 'left ventricular global longitudinal strain'/exp | 1159 |
| #2 | 'acute hypothyroidism':ti,ab,kw OR 'central hypothyroidism':ti,ab,kw OR 'central hypothyroidisms':ti,ab,kw OR 'hypothyreoidism':ti,ab,kw OR 'hypothyreosis':ti,ab,kw OR 'hypothyroidea':ti,ab,kw OR 'hypothyroidism':ti,ab,kw OR 'hypothyroidisms':ti,ab,kw OR 'hypothyroidosis':ti,ab,kw OR 'hypothyrosis':ti,ab,kw OR 'primary hypothyroidism':ti,ab,kw OR 'primary hypothyroidisms':ti,ab,kw OR 'secondary hypothyroidism':ti,ab,kw OR 'secondary hypothyroidisms':ti,ab,kw OR 'thyroid deficiency':ti,ab,kw OR 'thyroid gland failure':ti,ab,kw OR 'thyroid insufficiency':ti,ab,kw OR 'thyroid stimulating hormone deficiency':ti,ab,kw OR 'thyroid-stimulating hormone deficiencies':ti,ab,kw OR 'thyroid-stimulating hormone deficiency':ti,ab,kw OR 'tsh deficiencies':ti,ab,kw OR 'tsh deficiency':ti,ab,kw | 58703 |
| #1 | 'hypothyroidism'/exp | 91242 |

**Search strategy of Cochrane Library**

| NO. | Search deatiles | Hits |
| --- | --- | --- |
| #1 | MeSH descriptor: [Hypothyroidism] explode all trees | 746 |
| #2 | MeSH descriptor: [Global Longitudinal Strain] explode all trees | 3 |
| #3 | (acute hypothyroidism):ti,ab,kw OR (Central Hypothyroidism):ti,ab,kw OR (Central Hypothyroidisms):ti,ab,kw OR (hypothyreoidism):ti,ab,kw OR (hypothyreosis):ti,ab,kw OR (hypothyroidea):ti,ab,kw OR (hypothyroidism):ti,ab,kw OR (Hypothyroidisms):ti,ab,kw OR (hypothyroidosis):ti,ab,kw OR (hypothyrosis):ti,ab,kw OR (primary hypothyroidism):ti,ab,kw OR (Primary Hypothyroidisms):ti,ab,kw OR (Secondary Hypothyroidism):ti,ab,kw OR (Secondary Hypothyroidisms):ti,ab,kw OR (thyroid deficiency):ti,ab,kw OR (thyroid gland failure):ti,ab,kw OR (thyroid insufficiency):ti,ab,kw OR (Thyroid Stimulating Hormone Deficiency):ti,ab,kw OR (Thyroid-Stimulating Hormone Deficiencies):ti,ab,kw OR (Thyroid-Stimulating Hormone Deficiency):ti,ab,kw OR (TSH Deficiencies):ti,ab,kw OR (TSH Deficiency):ti,ab,kw | 3360 |
| #4 | (Global Longitudinal Strains):ti,ab,kw OR (Global Longitudinal Strain):ti,ab,kw OR (Left Ventricular Global Longitudinal Strain):ti,ab,kw OR (LV-GLS):ti,ab,kw OR (LV GLS):ti,ab,kw OR (global left ventricle longitudinal strain (GLS)):ti,ab,kw OR (left ventricle longitudinal strain):ti,ab,kw OR (left ventricle longitudinal strain (LV LS)):ti,ab,kw OR (left ventricular (LV) global longitudinal strain (GLS)):ti,ab,kw OR (left ventricular global longitudinal strain (LV-GLS)):ti,ab,kw OR (left ventricular global longitudinal strain (LVGLS)):ti,ab,kw OR (left ventricular GLS):ti,ab,kw OR (LV global longitudinal strain):ti,ab,kw OR (LV global longitudinal strain (GLS)):ti,ab,kw OR (LV longitudinal global strain (GLS)):ti,ab,kw OR (LV longitudinal global strain (LV-GLS)):ti,ab,kw | 710 |
| #5 | (Left ventricular strain):ti,ab,kw OR (Left ventricular deformation):ti,ab,kw OR (Left ventricular myocardial strain):ti,ab,kw OR (Myocardial deformation imaging):ti,ab,kw OR (Speckle tracking echocardiography):ti,ab,kw OR (Longitudinal strain):ti,ab,kw OR (Circumferential strain):ti,ab,kw OR (Radial strain):ti,ab,kw OR (3D strain):ti,ab,kw OR (Regional strain):ti,ab,kw OR (Strain rate):ti,ab,kw OR (Strain imaging):ti,ab,kw | 3658 |
| #6 | (#1 OR #3) AND (#2 OR #4 OR #5) | 6 |

**Search strategy of web of science**

| NO. | Search deatiles | Results |
| --- | --- | --- |
| #1 | ((((((((((((((((((((TS=(acute hypothyroidism) OR TS=(Central Hypothyroidism)) OR TS=(Central Hypothyroidisms)) OR TS=(hypothyreoidism)) OR TS=(hypothyreosis)) OR TS=(hypothyroidea)) OR TS=(hypothyroidism)) OR TS=(Hypothyroidisms)) OR TS=(hypothyroidosis)) OR TS=(hypothyrosis)) OR TS=(primary hypothyroidism)) OR TS=(Primary Hypothyroidisms)) OR TS=(Secondary Hypothyroidism)) OR TS=(Secondary Hypothyroidisms)) OR TS=(thyroid deficiency)) OR TS=(thyroid gland failure)) OR TS=(thyroid insufficiency)) OR TS=(Thyroid Stimulating Hormone Deficiency)) OR TS=(Thyroid-Stimulating Hormone Deficiencies)) OR TS=(Thyroid-Stimulating Hormone Deficiency)) OR TS=(TSH Deficiencies)) OR TS=(TSH Deficiency) | 44186 |
| #2 | ((((((((((((((TS=(Global Longitudinal Strains) OR TS=(Global Longitudinal Strain)) OR TS=(Left Ventricular Global Longitudinal Strain)) OR TS=(LV-GLS)) OR TS=(LV GLS)) OR TS=(global left ventricle longitudinal strain (GLS))) OR TS=(left ventricle longitudinal strain)) OR TS=(left ventricle longitudinal strain (LV LS))) OR TS=(left ventricular (LV) global longitudinal strain (GLS))) OR TS=(left ventricular global longitudinal strain (LV-GLS))) OR TS=(left ventricular global longitudinal strain (LVGLS))) OR TS=(left ventricular GLS)) OR TS=(LV global longitudinal strain)) OR TS=(LV global longitudinal strain (GLS))) OR TS=(LV longitudinal global strain (GLS))) OR TS=(LV longitudinal global strain (LV-GLS)) | 7944 |
| #3 | ((((((((((TS=(Left ventricular strain) OR TS=(Left ventricular deformation)) OR TS=(Left ventricular myocardial strain)) OR TS=(Myocardial deformation imaging)) OR TS=(Speckle tracking echocardiography)) OR TS=(Longitudinal strain)) OR TS=(Circumferential strain)) OR TS=(Radial strain)) OR TS=(3D strain)) OR TS=(Regional strain)) OR TS=(Strain rate)) OR TS=(Strain imaging) | 319270 |
| #4 | #3 OR #2 | 319292 |
| #5 | #1 AND #4 | 72 |

**
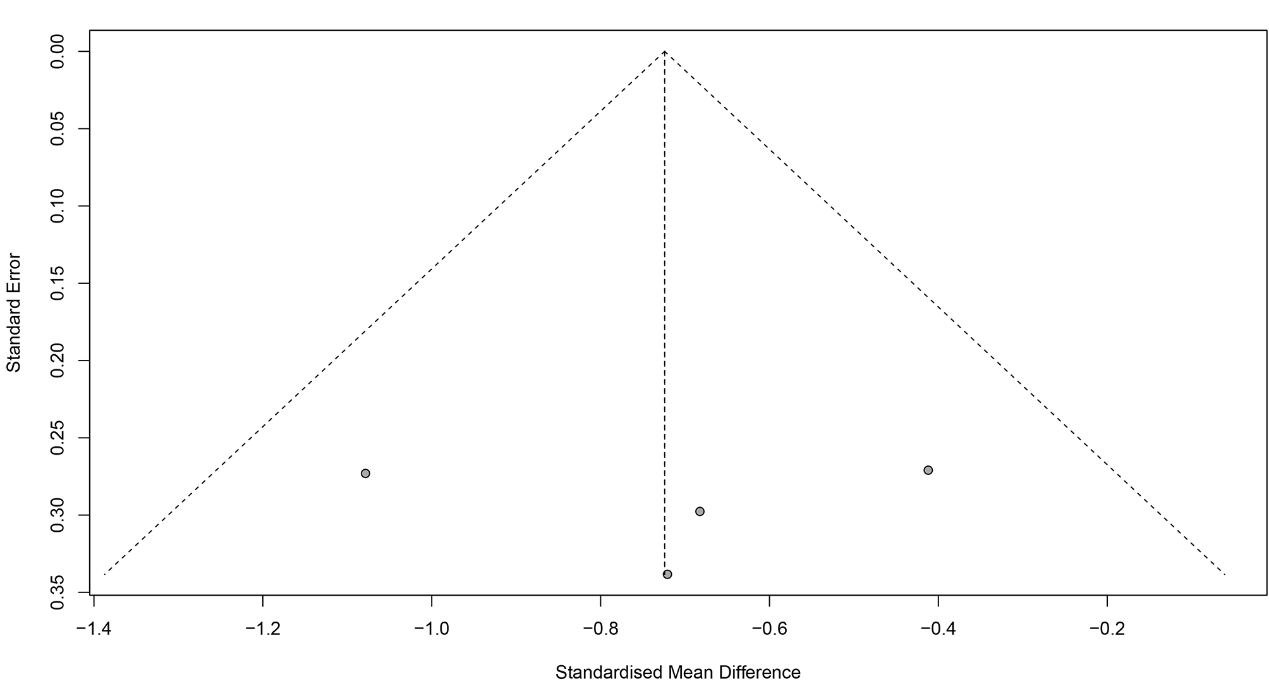
**

**Supplementary Figure 1** Funnel plot of early diastolic mitral flow velocity

**
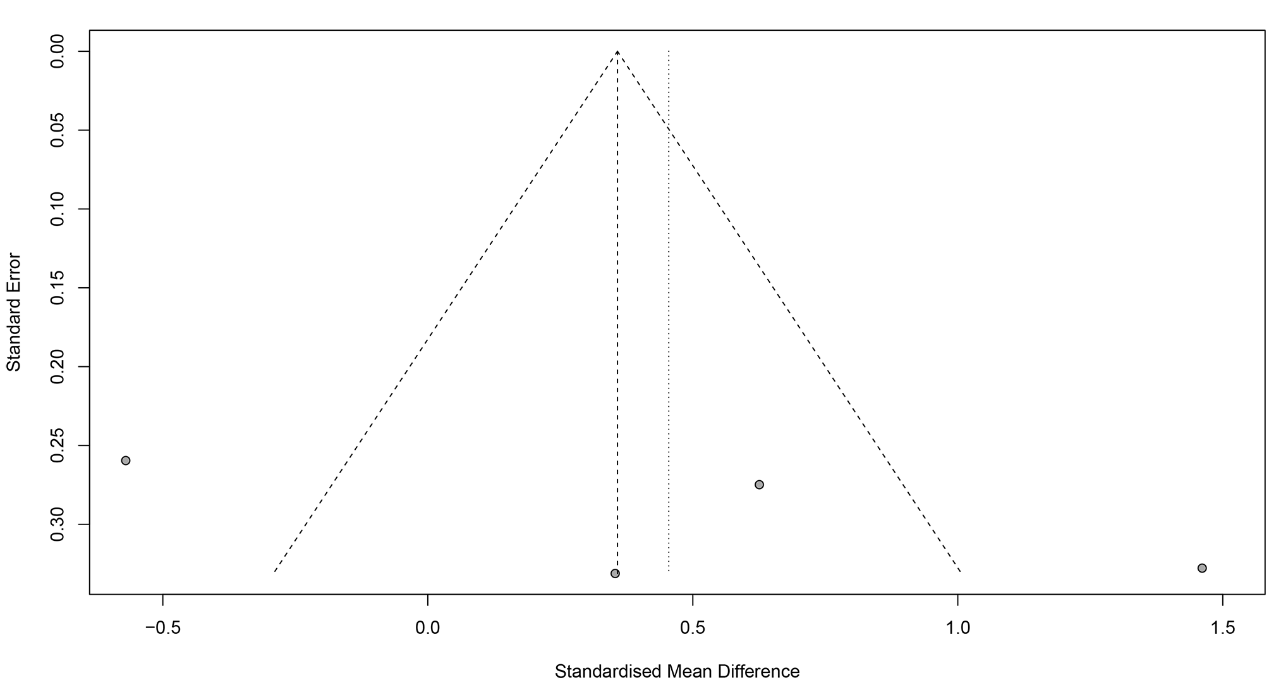
**

**Supplementary Figure 2** Funnel plot of late diastolic mitral flow velocity

**
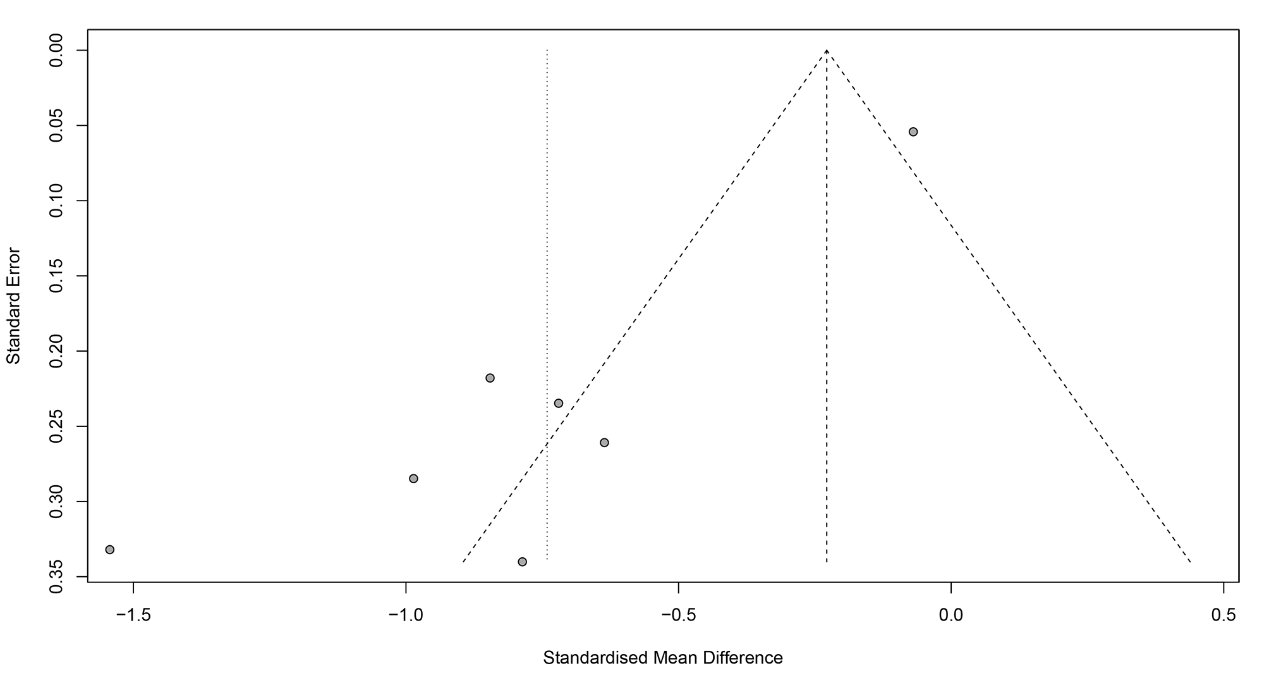
**

**Supplementary Figure 3** Funnel plot of early diastolic mitral flow velocity/late diastolic mitral flow velocity (E/A) ratio

**
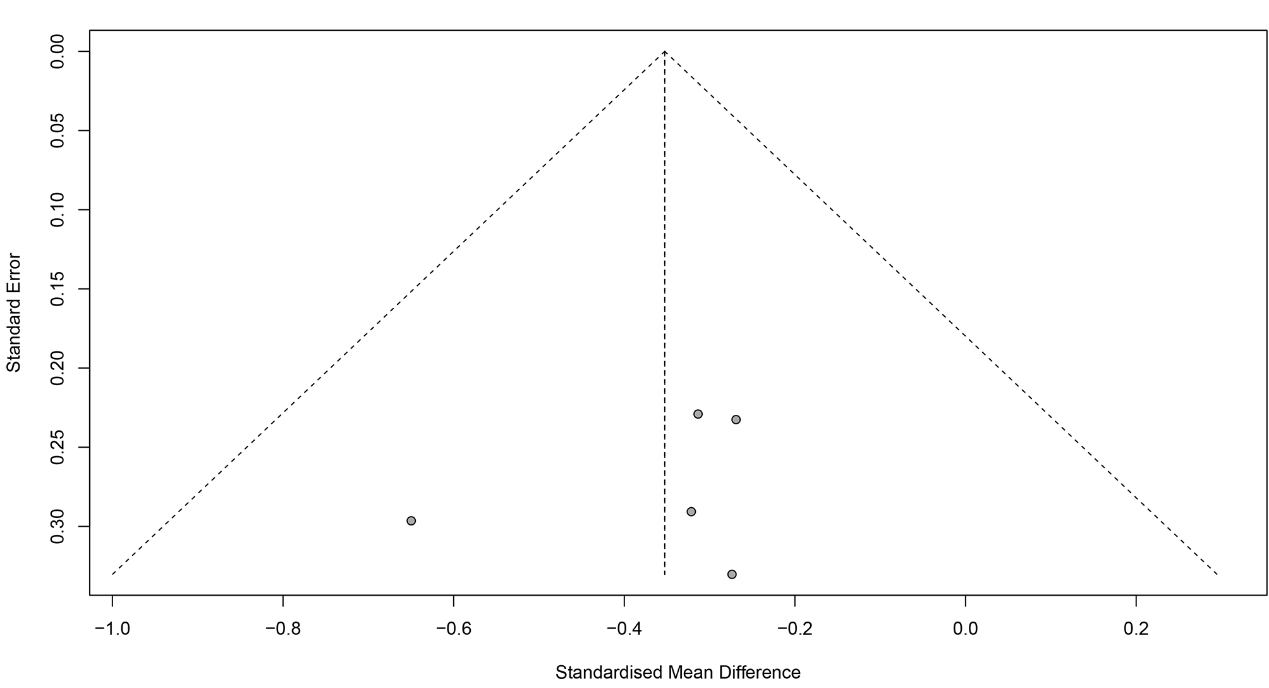
**

**Supplementary Figure 4** Funnel plot of ejection fraction


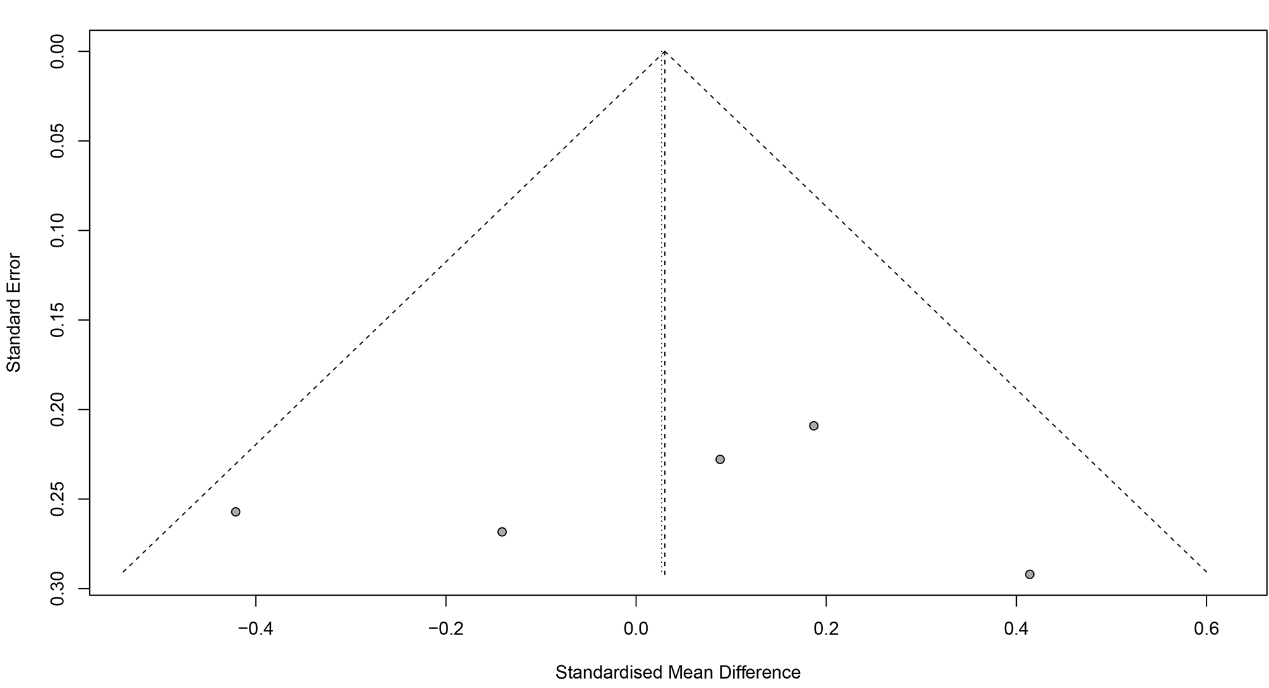


**Supplementary Figure 5** Funnel plot of end diastolic diameter


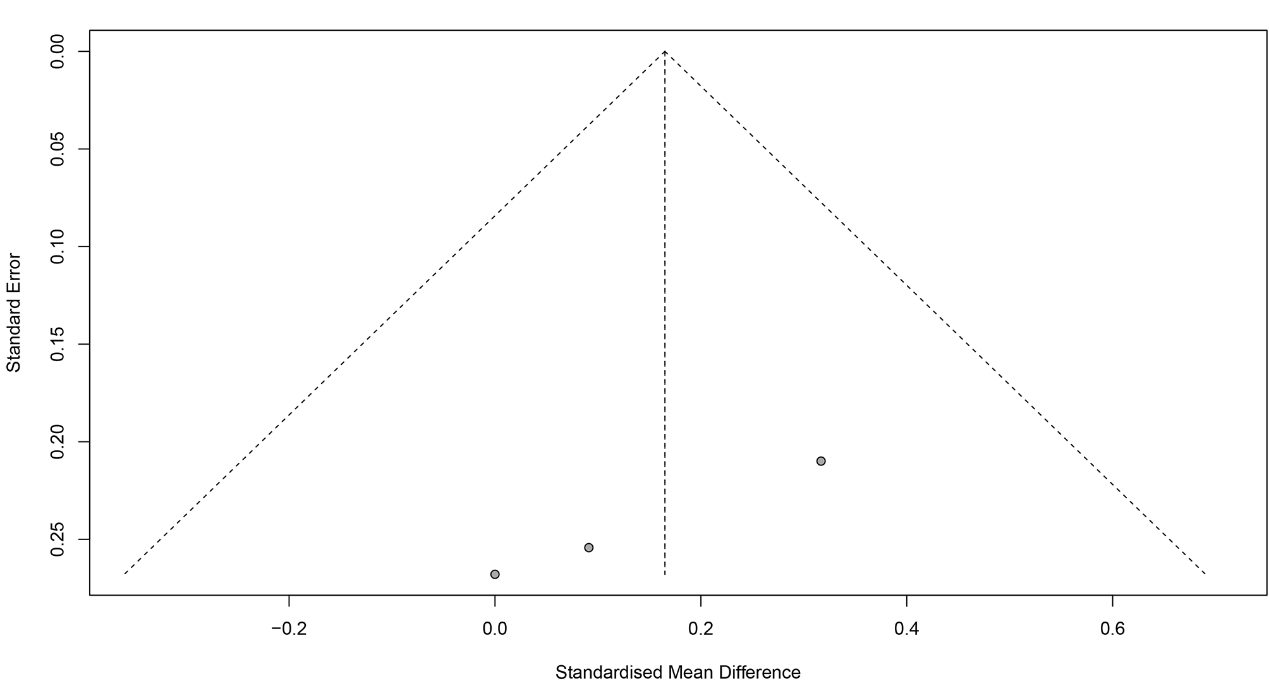


**Supplementary Figure 6** Funnel plot of end systolic diameter


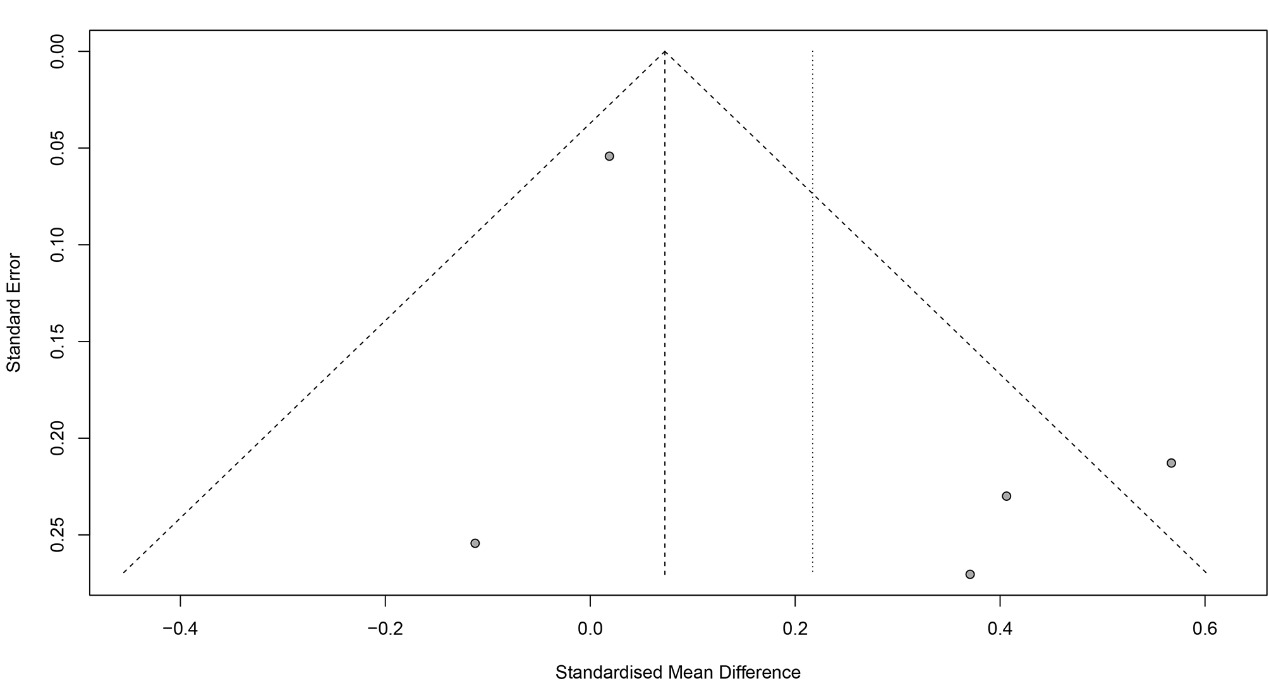


**Supplementary Figure 7** Funnel plot of diastolic left ventricle interventricular septum thickness


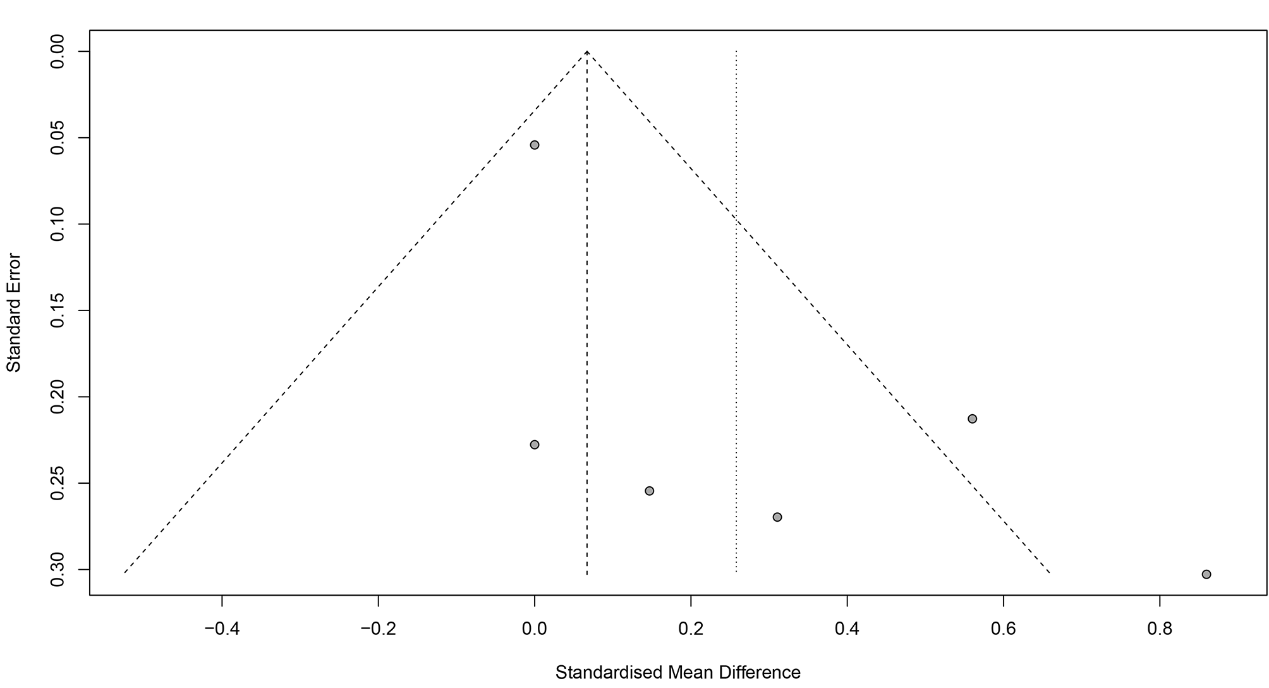


**Supplementary Figure 8** Funnel plot of diastolic left ventricle posterior wall thickness


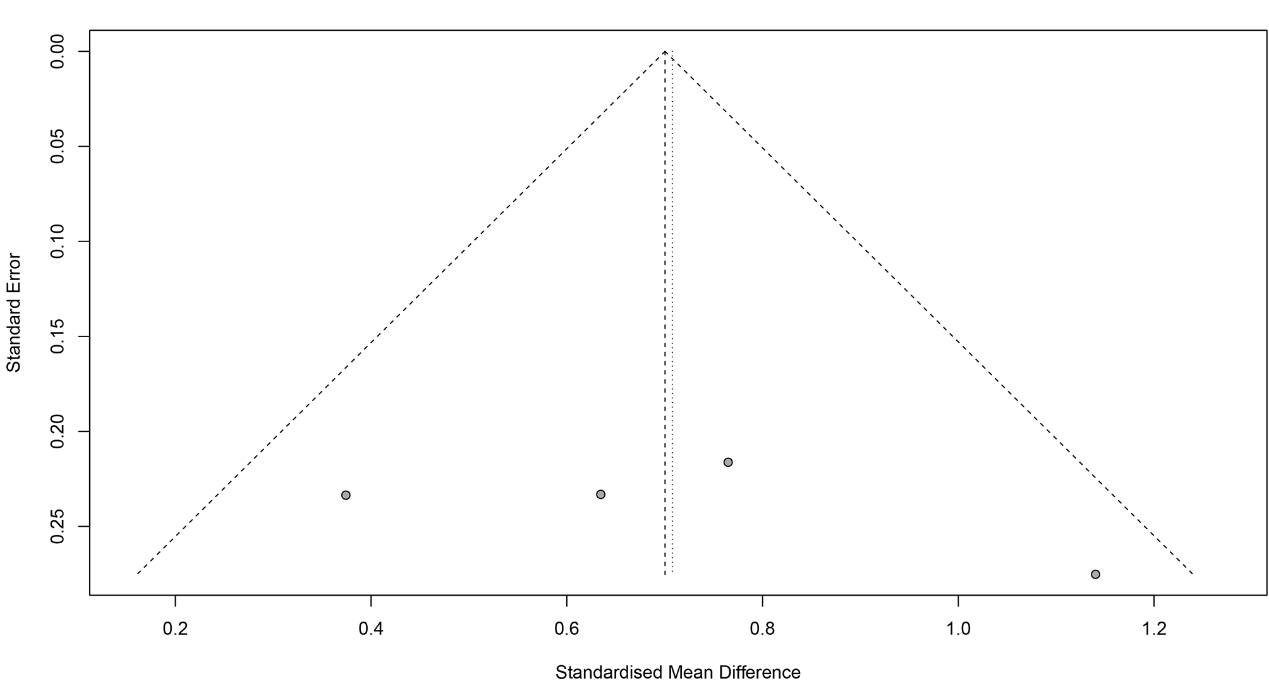


**Supplementary Figure 9** Funnel plot of global longitudinal strain


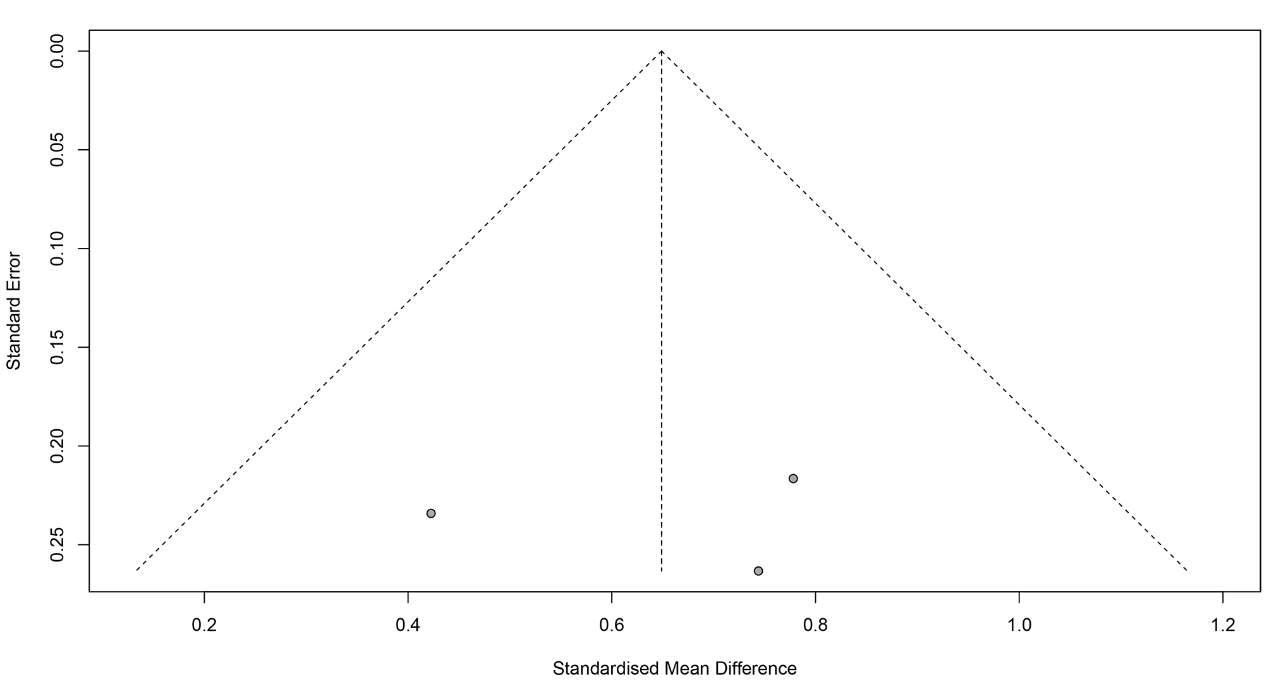


**Supplementary Figure 10** Funnel plot of global circumferential strain


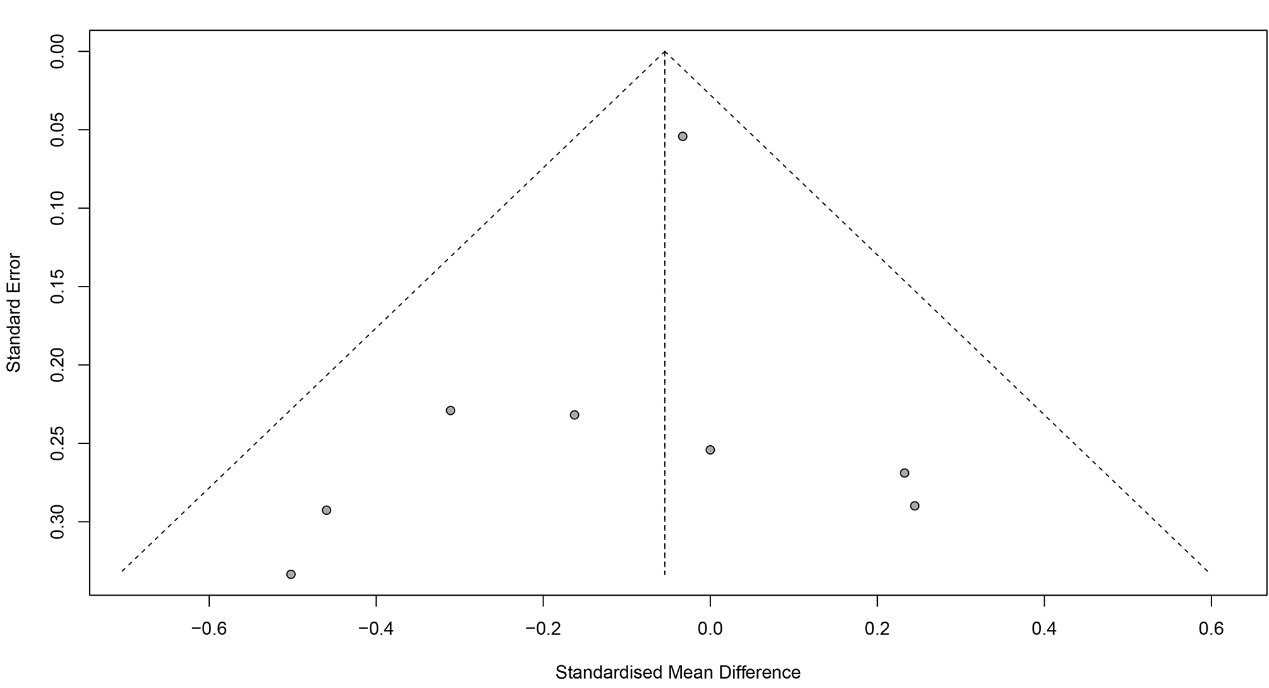


**Supplementary Figure 11** Funnel plot of heart rate


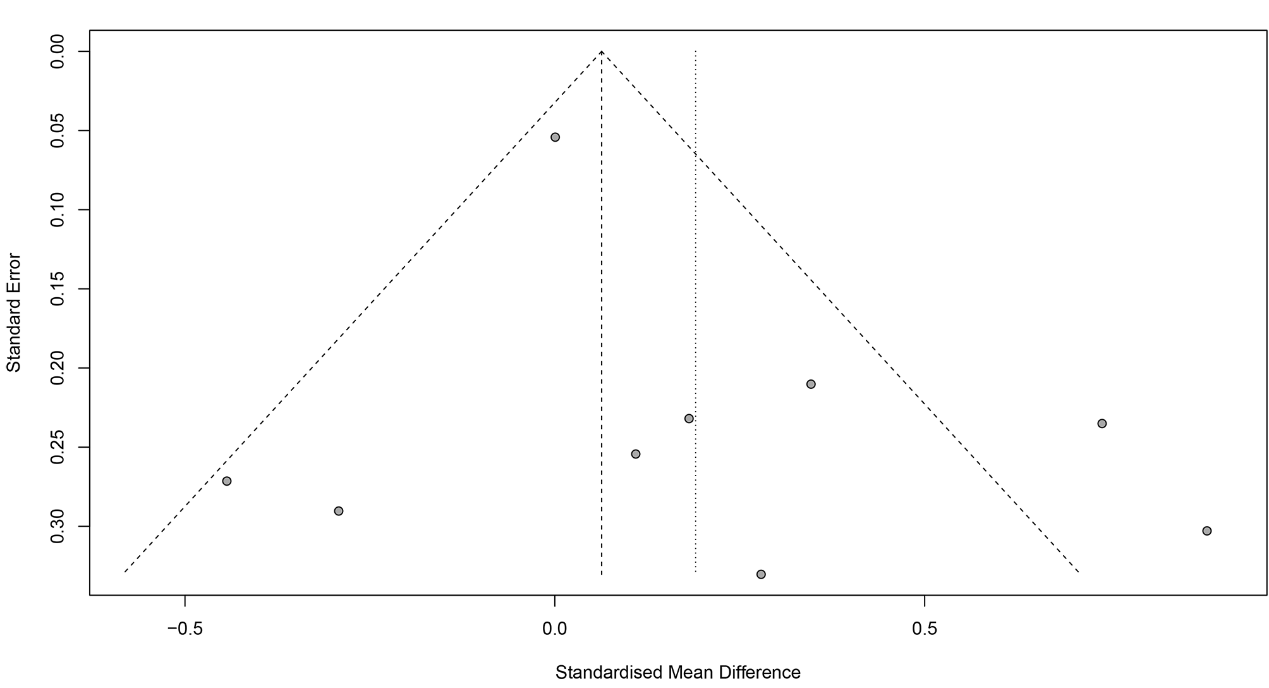


**Supplementary Figure 12** Funnel plot of systolic blood pressure


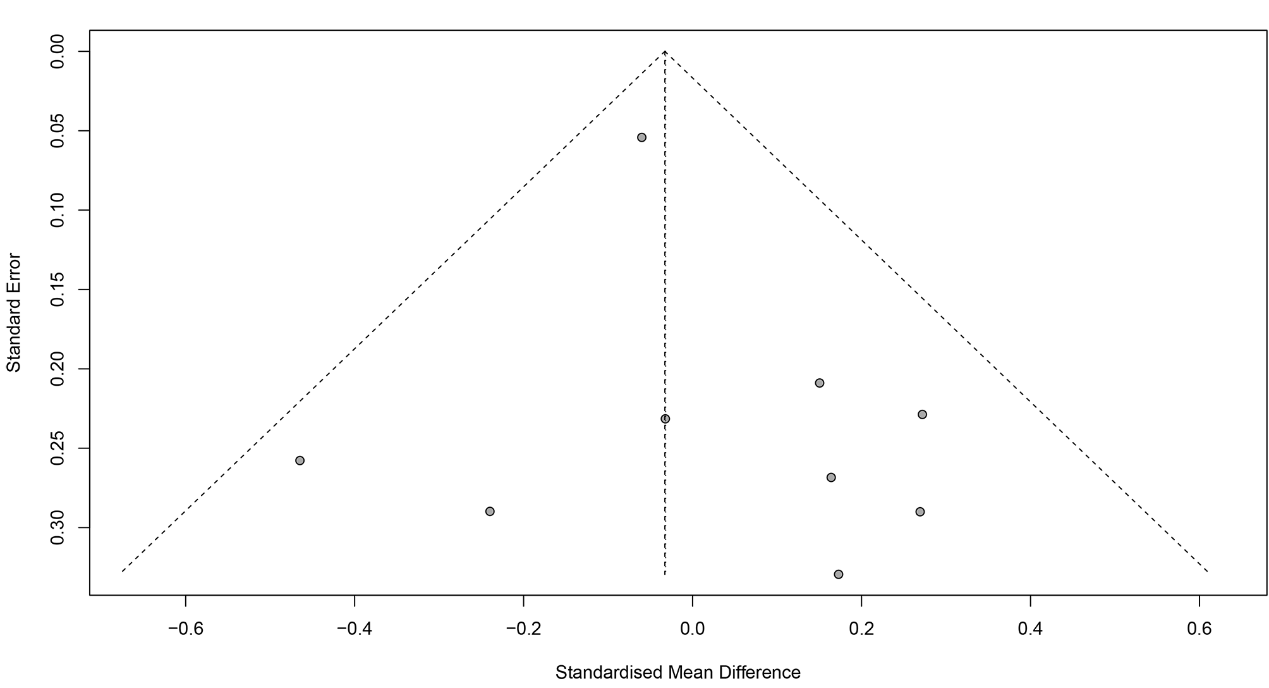


**Supplementary Figure 13** Funnel plot of diastolic blood pressure


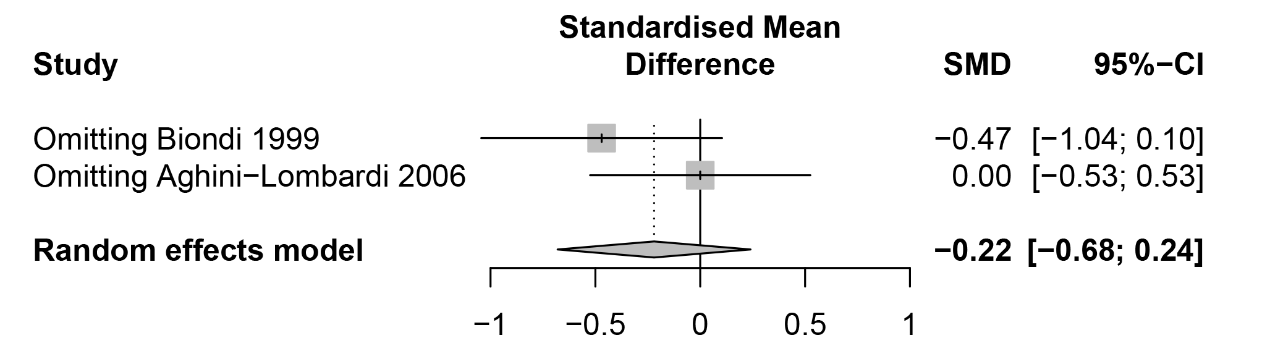


**Supplementary Figure 14** Forest plot of sensitivity analysis on fractional shortening

**
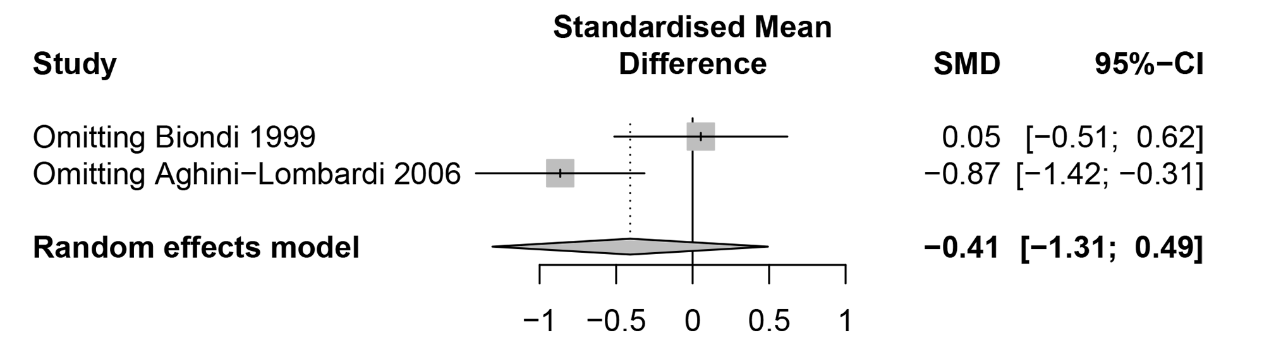
**

**Supplementary Figure 15** Forest plot of sensitivity analysis on systemic vascular resistance

**
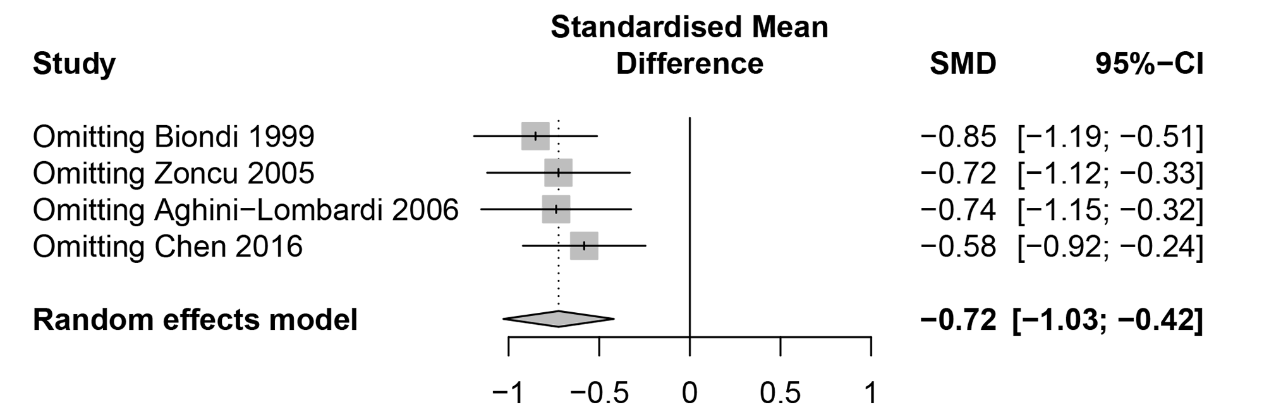
**

**Supplementary Figure 16** Forest plot of sensitivity analysis on early diastolic mitral flow velocity

**
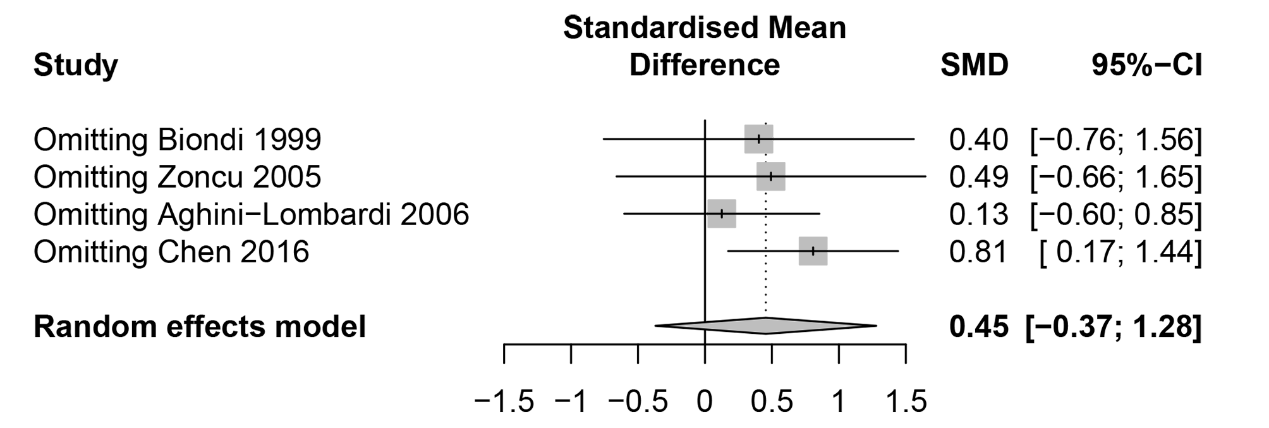
**

**Supplementary Figure 17** Forest plot of sensitivity analysis on late diastolic mitral flow velocity

**
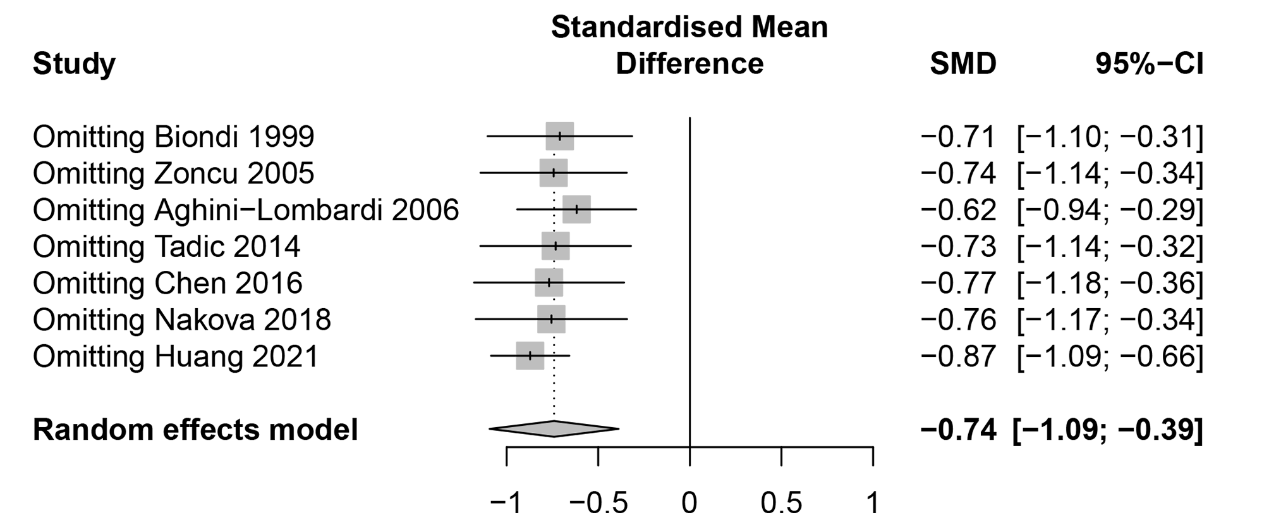
**

**Supplementary Figure 18** Forest plot of sensitivity analysis on early diastolic mitral flow velocity/late diastolic mitral flow velocity (E/A) ratio

**
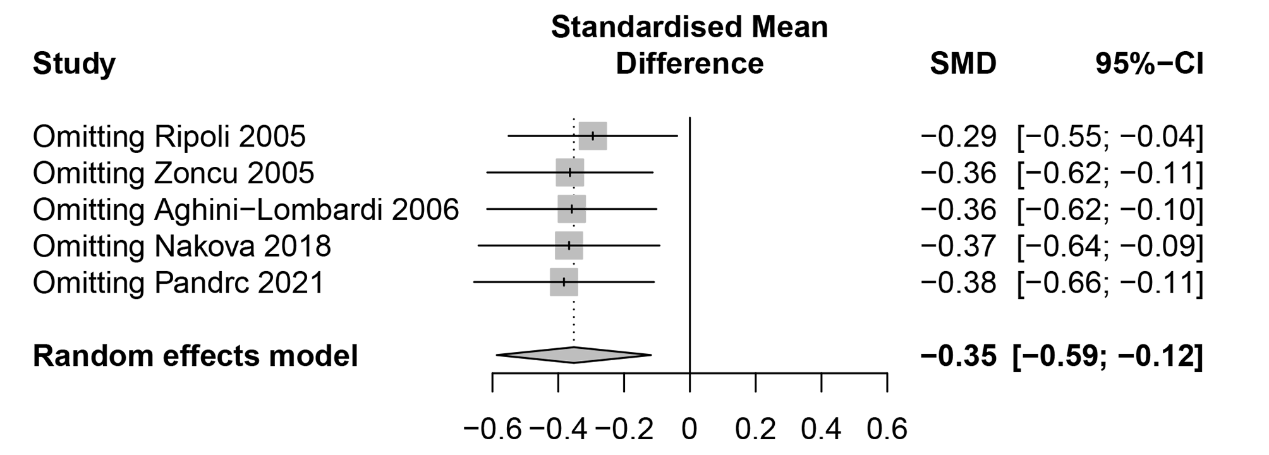
**

**Supplementary Figure 19** Forest plot of sensitivity analysis on of ejection fraction


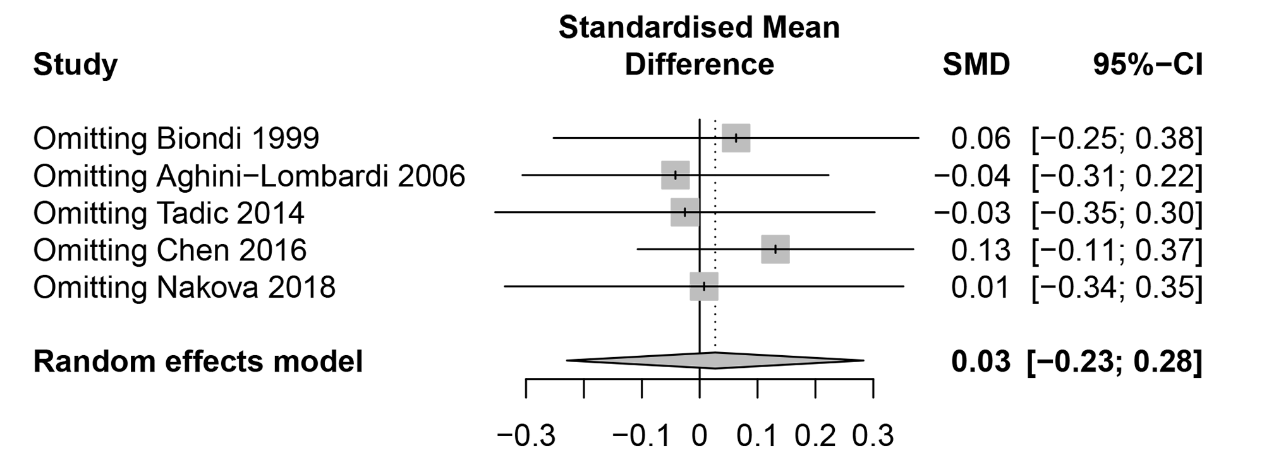


**Supplementary Figure 20** Forest plot of sensitivity analysis on end diastolic diameter


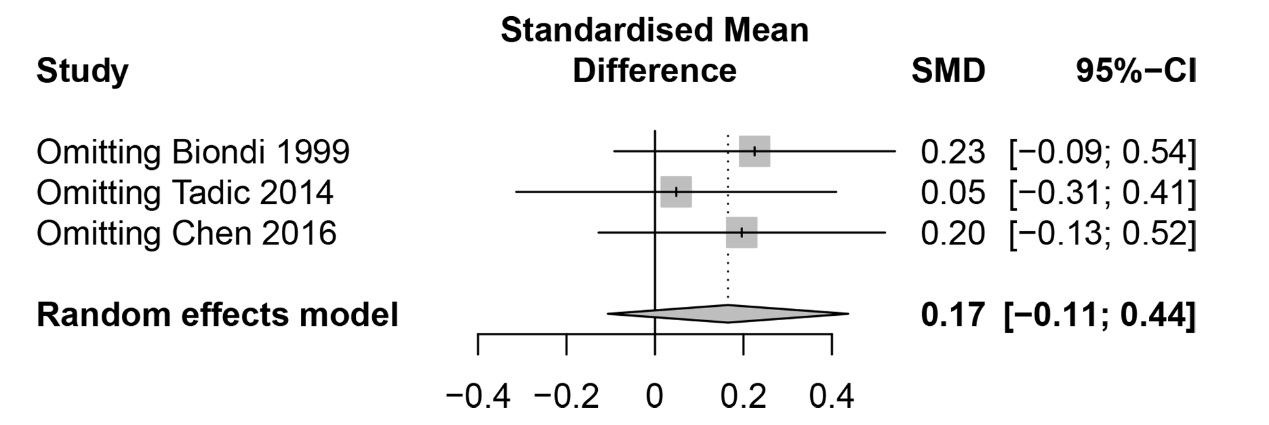


**Supplementary Figure 21** Forest plot of sensitivity analysis on systolic diameter


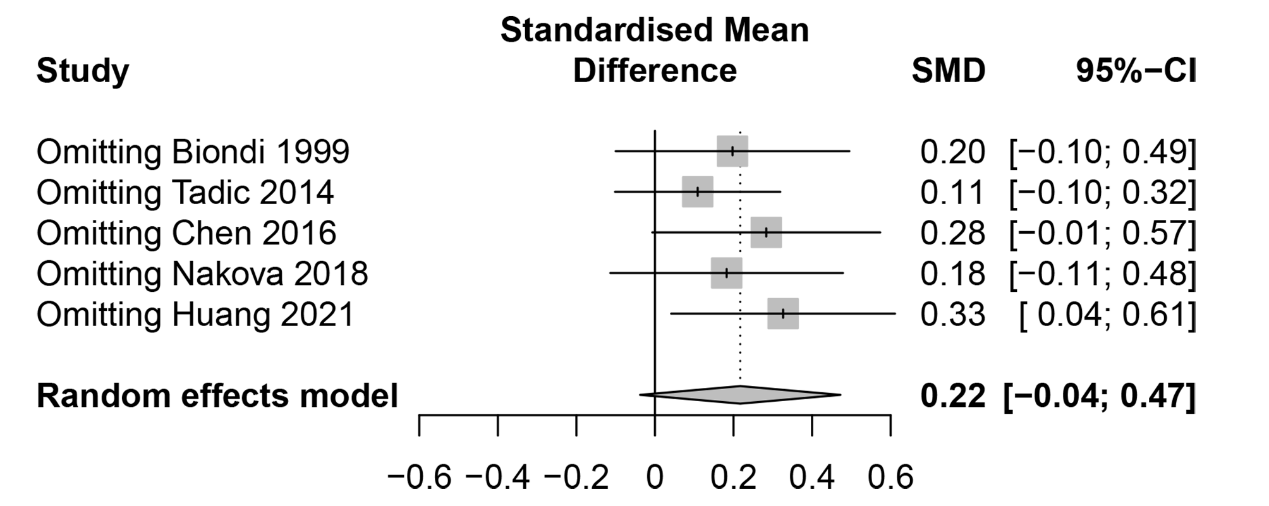


**Supplementary Figure 22** Forest plot of sensitivity analysis on diastolic left ventricle interventricular septum thickness


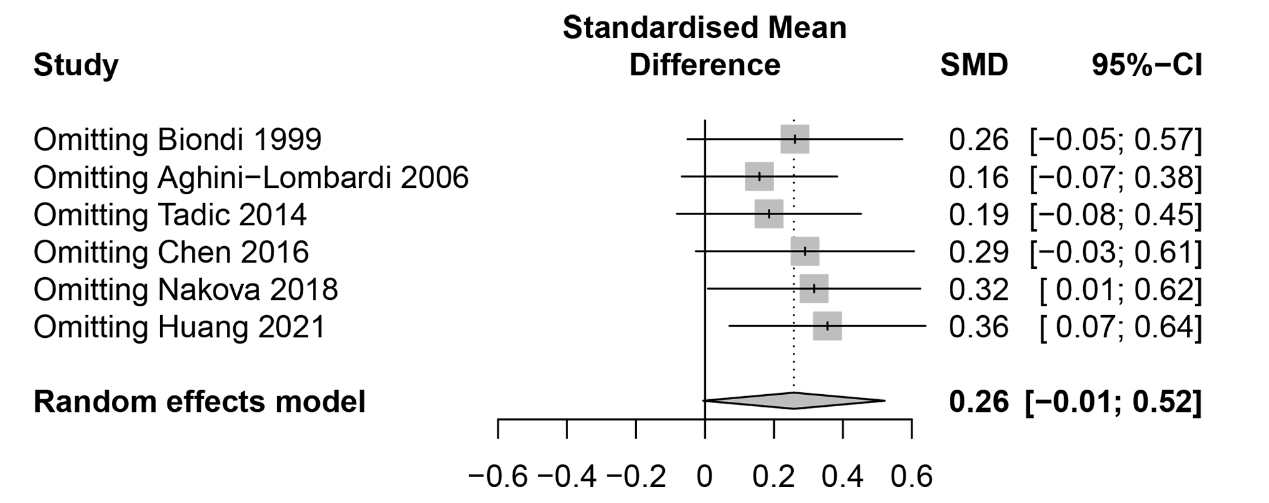


**Supplementary Figure 23** Forest plot of sensitivity analysis on diastolic left ventricle posterior wall thickness


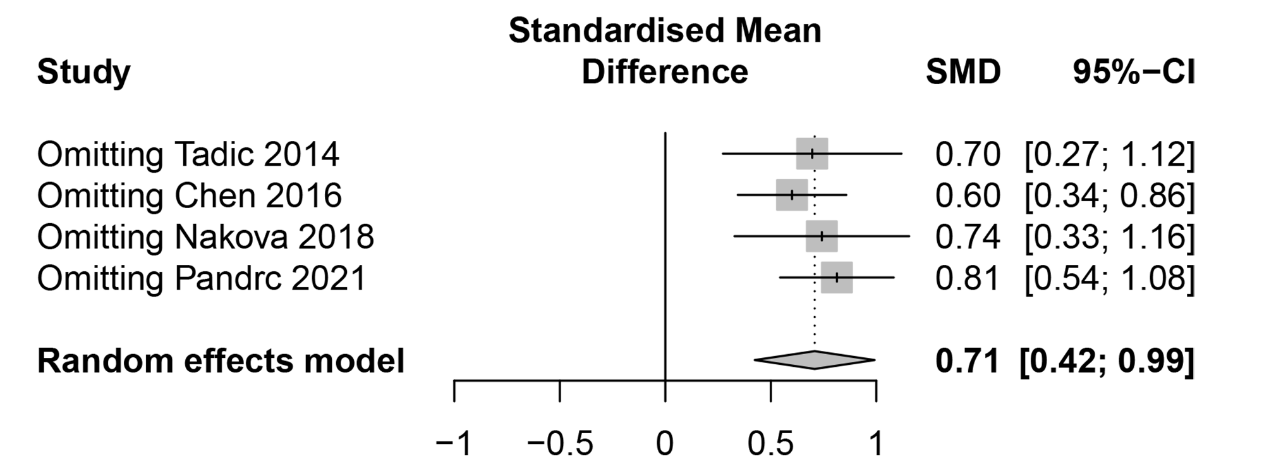


**Supplementary Figure 24** Forest plot of sensitivity analysis on global longitudinal strain


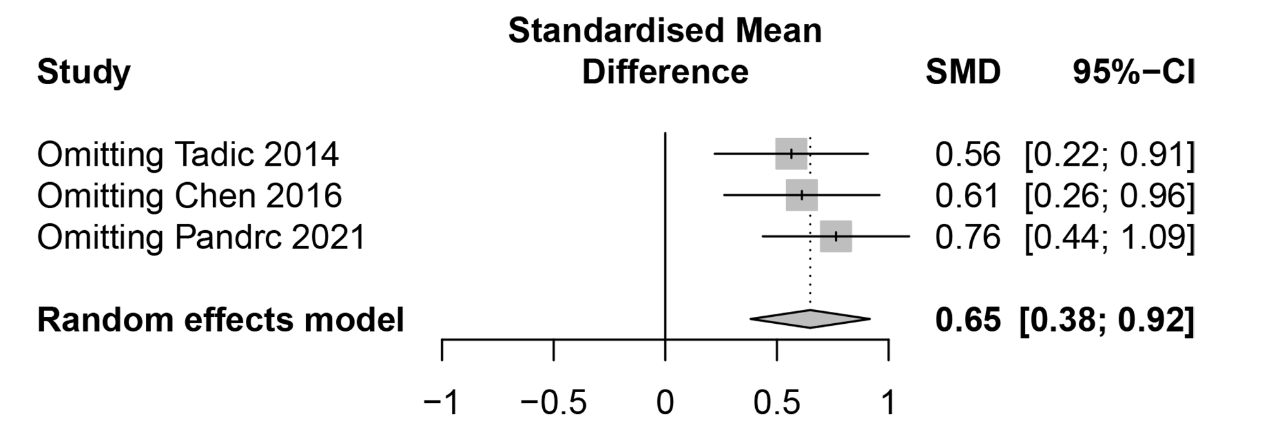


**Supplementary Figure 25** Forest plot of sensitivity analysis on global circumferential strain


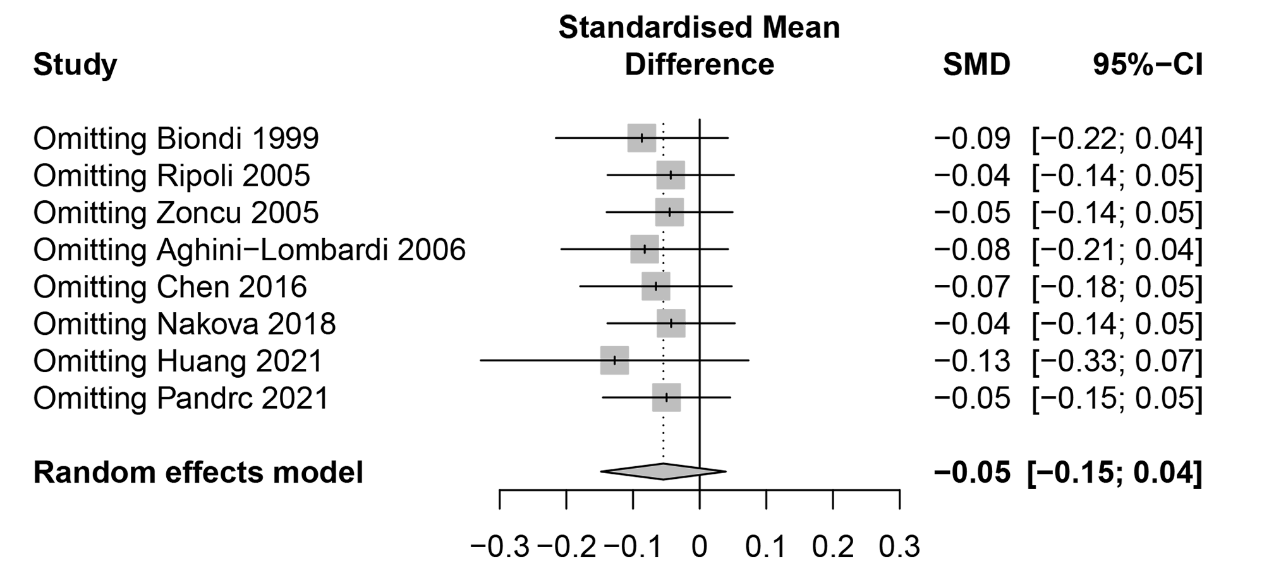


**Supplementary Figure 26** Forest plot of sensitivity analysis on heart rate


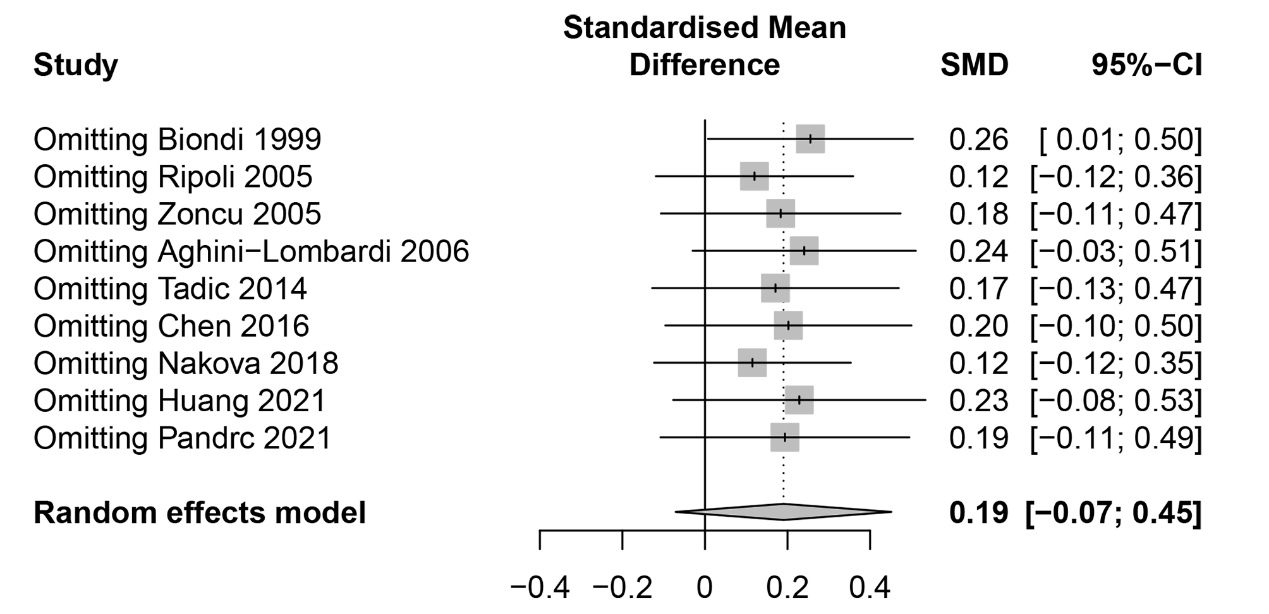


**Supplementary Figure 27** Forest plot of sensitivity analysis on systolic blood pressure


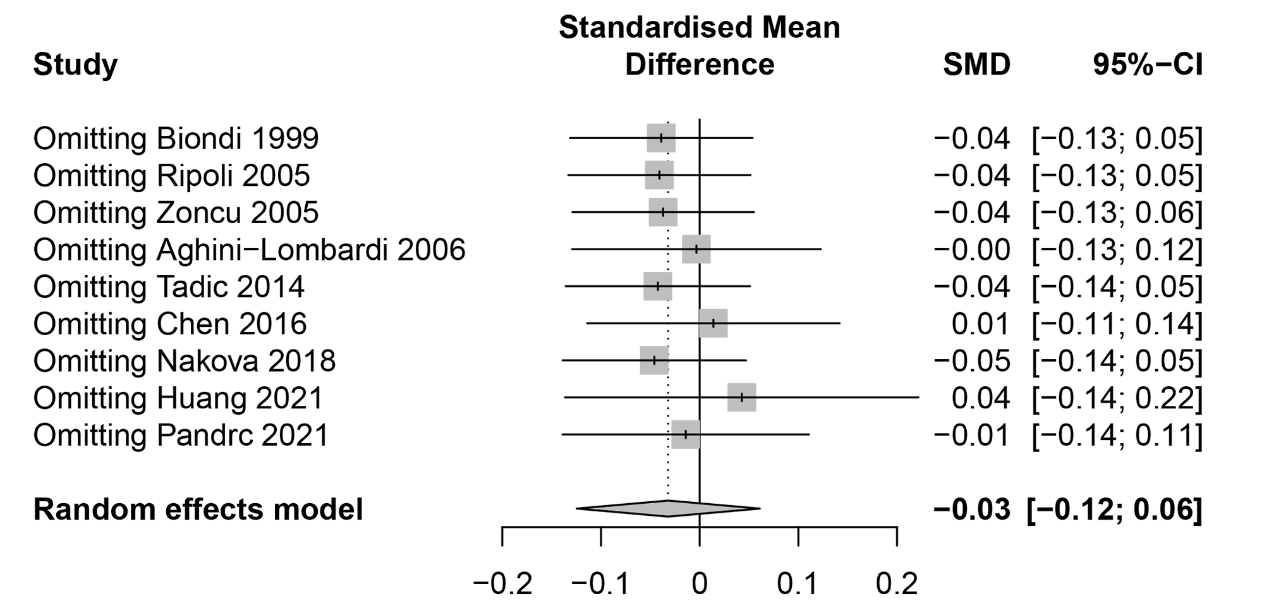


**Supplementary Figure 28** Forest plot of sensitivity analysis on diastolic blood pressure

**
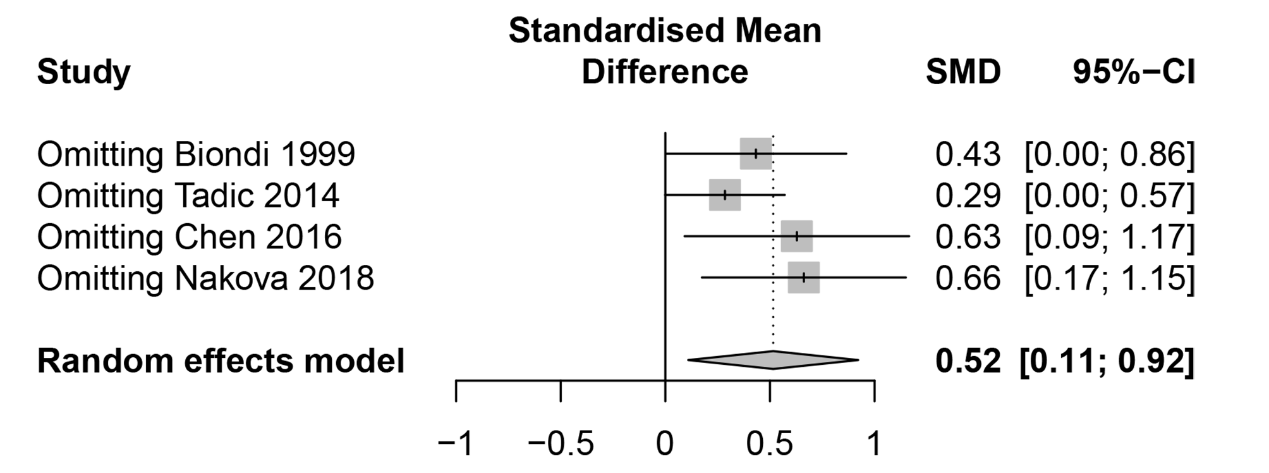
**

**Supplementary Figure 29** Forest plot of sensitivity analysis on early diastolic mitral flow velocity/late diastolic mitral flow velocity (E/A) ratio before vs. after treatment

**
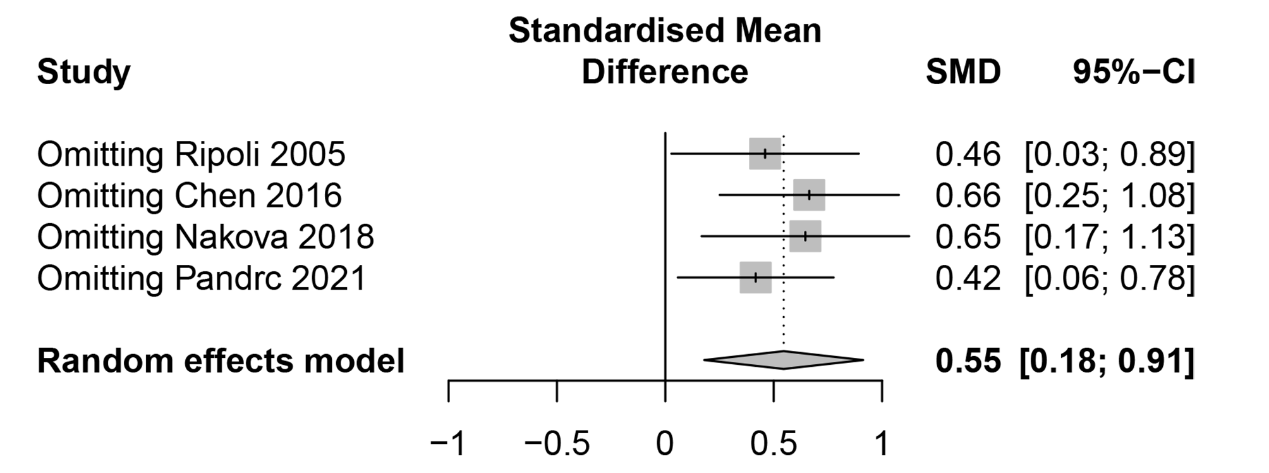
**

**Supplementary Figure 30** Forest plot of sensitivity analysis on of ejection fraction before vs. after treatment


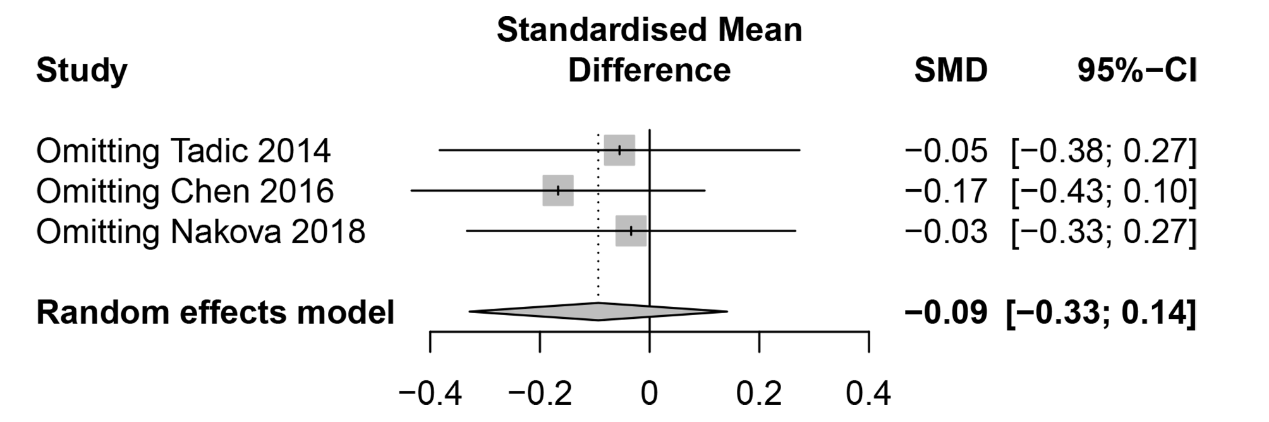


**Supplementary Figure 31** Forest plot of sensitivity analysis on end diastolic diameter before vs. after treatment


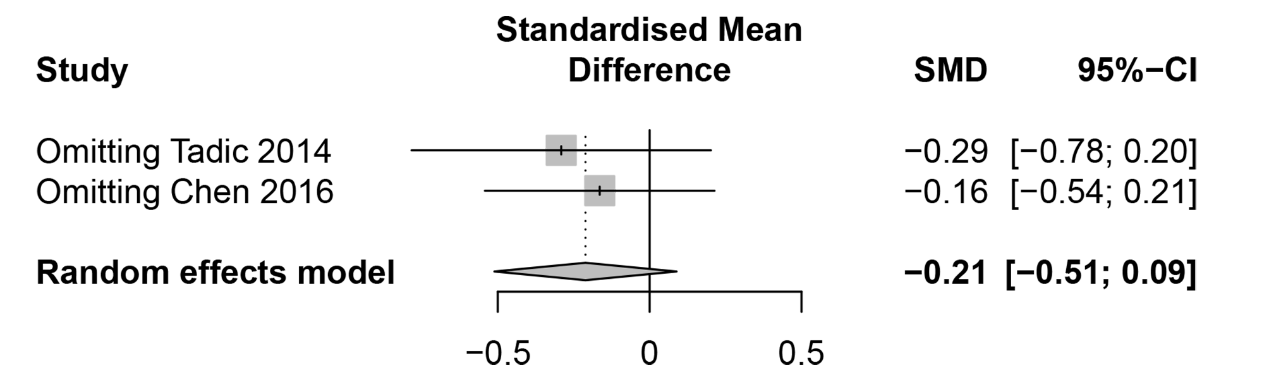


**Supplementary Figure 32** Forest plot of sensitivity analysis on systolic diameter before vs. after treatment


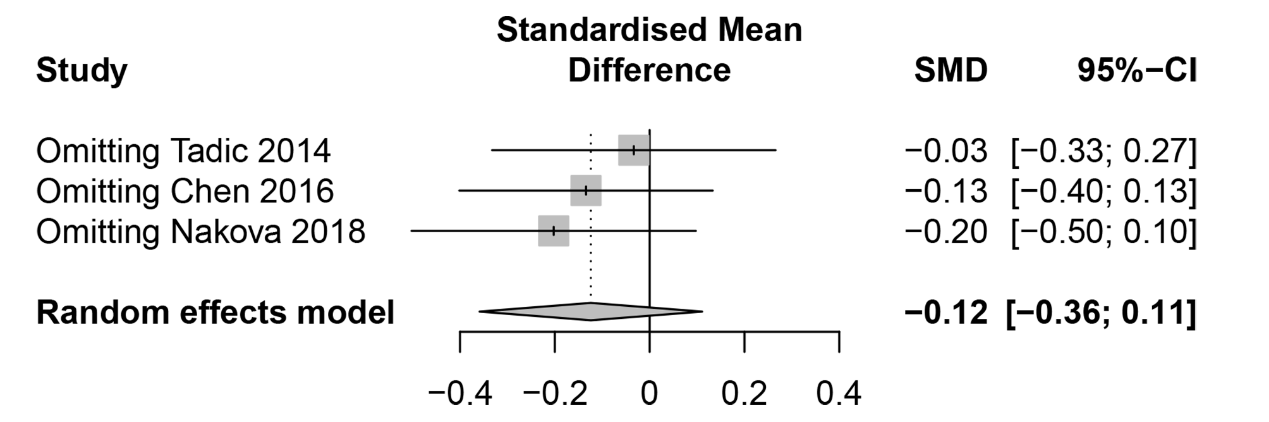


**Supplementary Figure 33** Forest plot of sensitivity analysis on diastolic left ventricle interventricular septum thickness before vs. after treatment


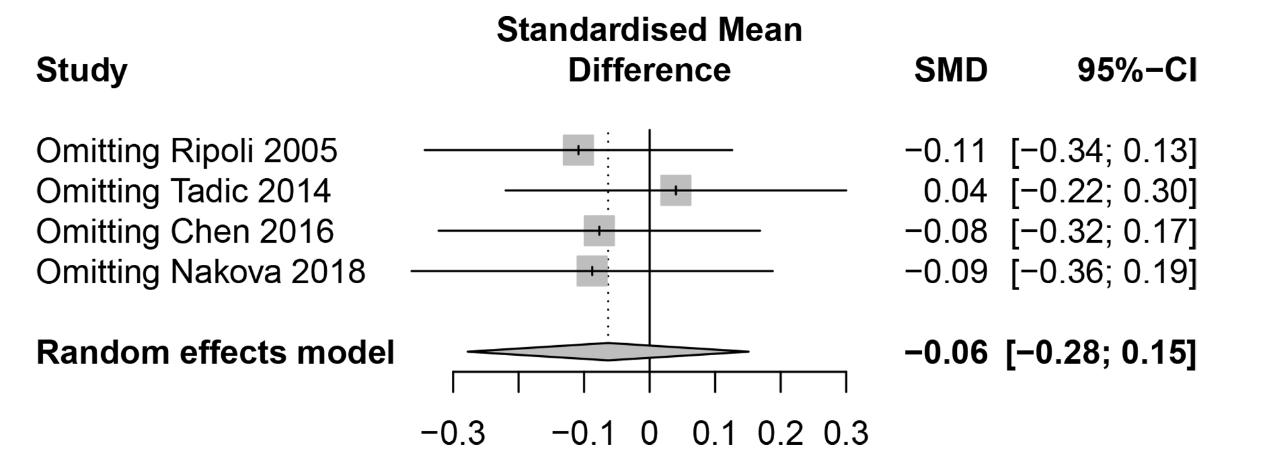


**Supplementary Figure 34** Forest plot of sensitivity analysis on diastolic left ventricle posterior wall thickness before vs. after treatment


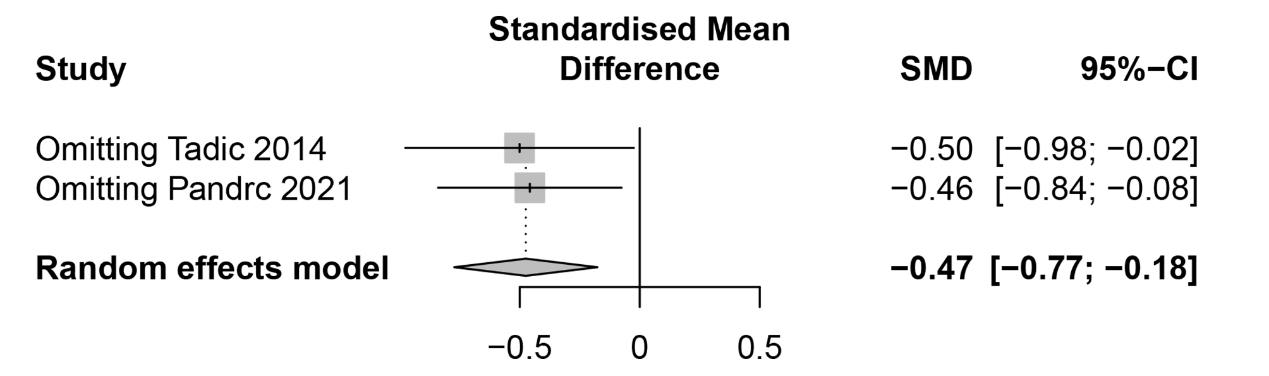


**Supplementary Figure 35** Forest plot of sensitivity analysis on global longitudinal strain before vs. after treatment


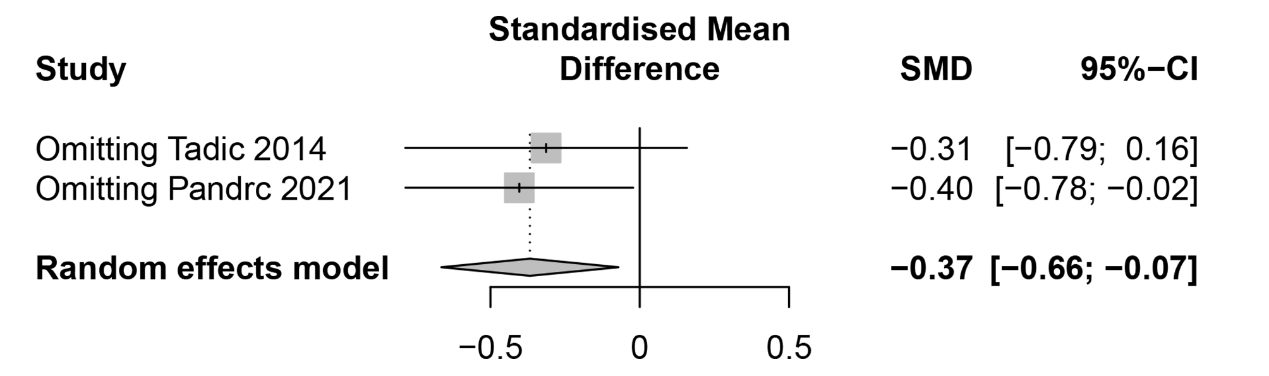


**Supplementary Figure 36** Forest plot of sensitivity analysis on global circumferential strain before vs. after treatment
